# De Novo Transcriptome Assembly and Functional Annotation in Five Species of Bats

**DOI:** 10.1038/s41598-019-42560-9

**Published:** 2019-04-17

**Authors:** Diana D. Moreno-Santillán, Carlos Machain-Williams, Georgina Hernández-Montes, Jorge Ortega

**Affiliations:** 1 Escuela Nacional de Ciencias Biológicas, Posgrado Químicobiológicas, Instituto Politécnico Nacional, Departamento de Zoología, Ciudad de México, CDMX, Mexico; 2 Centro de Investigaciones Regionales Dr. Hideyo Noguchi, Universidad Autónoma de Yucatán, Laboratorio de Arbovirología, Mérida, Yucatán, Mexico; 3 Universidad Nacional Autónoma de México, Red de Apoyo a la Investigación, Ciudad de México, CDMX, Mexico

**Keywords:** RNA sequencing, Transcriptomics

## Abstract

High-throughput RNA sequencing is a powerful tool that allows us to perform gene prediction and analyze tissue-specific overexpression of genes, but also at species level comparisons can be performed, although in a more restricted manner. In the present study complete liver transcriptomes of five tropical bat species were *De novo* assembled and annotated. Highly expressed genes in the five species were involved in glycolysis and lipid metabolism pathways. Cross-species differential expression analysis was conducted using single copy orthologues shared across the five species. Between 22 and 29 orthologs were upregulated for each species. We detected upregulated expression in *Artibeus jamaicensis* genes related to fructose metabolism pathway. Such findings can be correlated with *A. jamaicensis* dietary habits, as it was the unique frugivorous species included. This is the first report of transcriptome assembly by RNA-seq in these species, except for *A. jamaicensis* and as far as our knowledge is the first cross-species comparisons of transcriptomes and gene expression in tropical bats.

## Introduction

The order Chiroptera is the second largest order of mammals and is divided into: two suborders: Yinpterochiroptera and Yangochiroptera^1^. Its diversity includes and estimated ~1,331 species distributed throughout the world, except for the polar regions and isolated islands. Bats present a wide diversity of feeding habits and may be carnivorous, frugivorous, hematophagous, insectivorous or nectarivorous^2^; as a consequence, chiropters play a crucial roles in the maintenance of the ecosystem balance by providing important ecological services; two-thirds of bats species are insectivorous and as such are considered biological pests controls of agricultural importance^2^. Similar to birds and insects, nectarivores act as pollinators and contribute to genetic exchange between flowering plants by pollination^2^. Frugivorous species play a crucial role in the renovation of disturbed landscapes and in the maintenance of vegetation within the ecosystem due to seed dispersion^2^. The success of bats in a wide diversity of niches is a result of evolutionary processes that have resulted in the appearance of diverse adaptations such as echolocation and ability to fly, which are conspicuous characteristics of bats.

Molecular studies using next generation sequencing have made it possible to analyse the whole genome and transcriptome of bats species and have contributed to a better understanding of these evolutionary processes as well as to a new taxonomic classification. For instance, molecular research has led to the rearrangement of Chiroptera phylogeny, bringing a new classification dividing the order into the two suborders Yinpterochiroptera and Yangochiroptera^1,3^ based on the observation that the Microchiroptera constitute a paraphyletic clade instead of a monophyletic group as previously thought^4^.

High-throughput sequencing is not only useful in phylogenetics, but also helps to reveal evolutionary and adaptative evolution in bats, such as the correlation between the increment in energy metabolism demand with the evolution of true self-powered flight, which is conspicuous in bats^5^. Whole-genome and transcriptome sequencing studies have also generate new findings regarding the bat immune system response^6^. Transcriptome analysis in the Australian flying fox (*P. alecto*)^7,8^ has provided insights into the co-evolution of bats and viruses by contributing to understanding the evolution of the chiropteran immune system and the apparent immunity or greater resistance of bats to viral infection in comparison with other mammals^9^. The study of the whole genome and transcriptome can unveil knowledge about evolution insights as well as ecological interactions in bats. Transcriptomic analysis allows the identification and discovery of novel transcripts and the quantification of differential tissue-specific gene expression. Consequently, the quantity of available bats’ transcriptomic resources for bat species has increased exponentially in recent years as the transcriptomes of several bats organs and tissues such as brain^10,11^, heart^10^, kidney^10,12^, liver^10^, lung^10,12^, lymph^10^, spleen^7,10,12^ and thymus^7^ have been sequenced and assembled. Also, transcriptomic data has been obtained from blood^13^ and wing biopsies^14^, avoiding the sacrifice of organisms when a tissue-specific transcriptomic analysis is not required. In this study, we aim to contribute to the currently available transcriptomic resources for bats by sequencing and assembling the complete liver transcriptomes of five bat species classified into five extant Chiroptera families, namely, *Artibeus jamaicensis* (Phyllostomidae), *Mormoops megalophylla* (Mormoopidae), *Myotis keaysi* (Vespertilionidae), *Nyctinomops laticaudatus* (Molossidae) and *Peropteryx macrotis* (Emballonuridae). We performed high-throughput sequencing of transcriptomes by RNA-sequencing (RNA-seq) and *de novo* assembly with Trinity because we are studying non-model organisms. RNA-seq is a powerful method for measuring transcriptome composition and to discovering putative new exons, as well as for understanding how genes are expressed in a species.

In this study we compared the transcriptomes of five species and performed differential expression analysis based only on orthologous transcripts, with the aim of analysing which genes, if any, are upregulated and downregulated in these species and to determine whether this transcript expression can be correlated with the biology of each species, such as dietary habits.

## Results and Discussion

### RNA-seq library construction and sequencing

**Figure 1:**
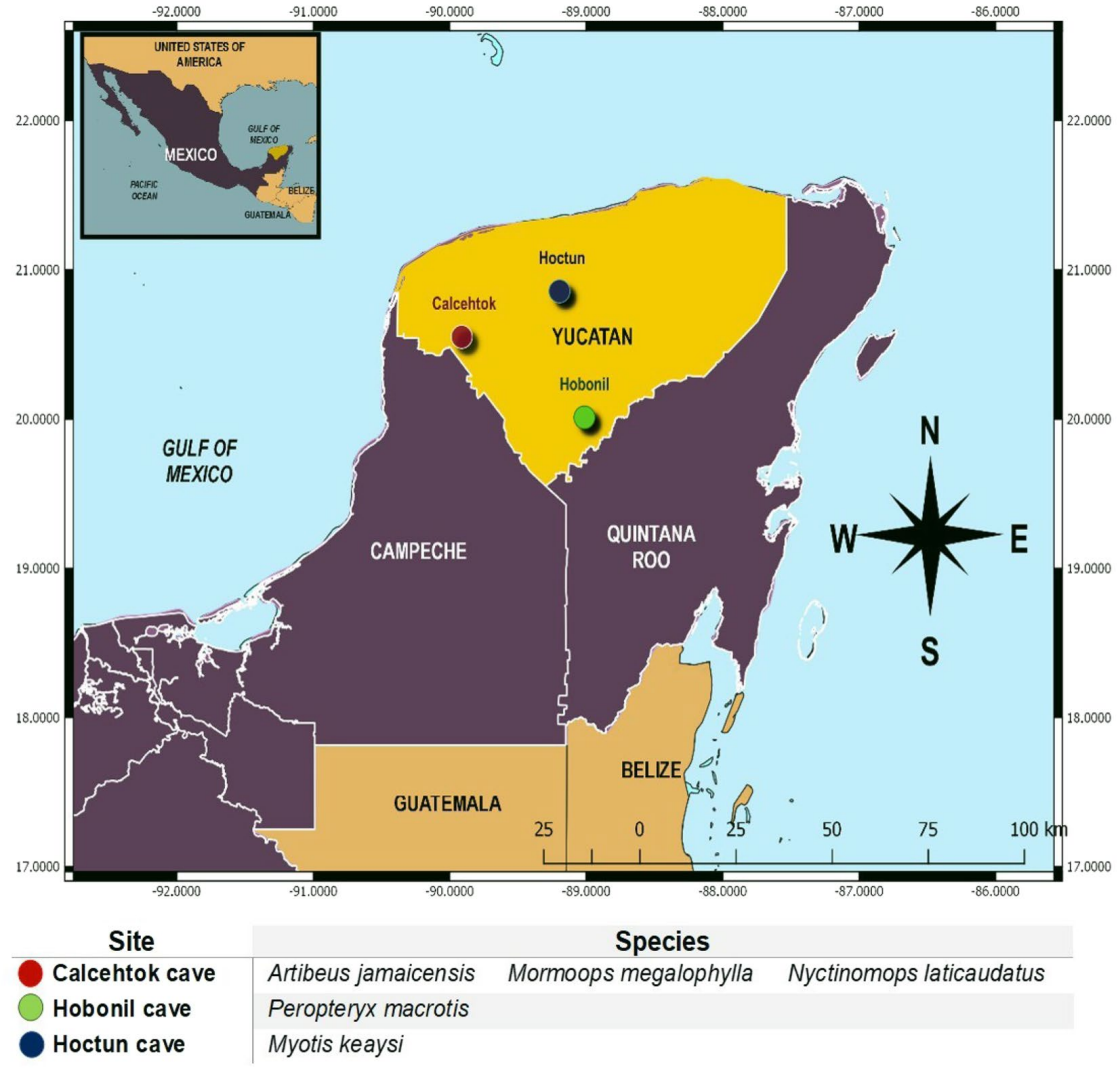
Sampling sites for the five species of bats from Yucatan Mexico. Three individuals were collected per specie. Three individuals of each species were collected. The map was generated using QGIS v.3.0.2 software^35^. Copyright © 2018 Moreno Diana.

Total RNA was extracted from liver tissue obtained from three biological replicates of each of five bats species (Table 1). All specimens were collected from three locations at Yucatan State in the south-eastern Mexico (Fig. 1). The RNA integrity numbers (RIN values) considered for library preparation ranged from 6.5 and 8. The mean size of the fifteen cDNA libraries was 360 bp. HiSeq. 4000 multiplex sequencing generated a total of 403 million paired-end reads of 101 bp length. For each individual sequencing, the depth ranged between 30 to 69 million paired end reads (Table 1). After quality filtering with cutadapt^15^ software, 99% of paired-end reads were conserved; these reads had final lengths between 30 and 101 bp and quality scores ≥ 30 (Table 1). A high proportion of the reads was retained after quality trimming, suggesting that although library construction was not performed using the traditional sample preparation kit manufactured by Illumina, but instead with the KAPA Stranded RNA-Seq with RiboErase kit from KapaBiosystems®, we were able to obtain libraries of sufficient quality for an accurate sequencing, and therefore a high coverage of good-quality reads, suggesting that the use of an alternative kit for RNA-seq library construction in non-model organisms was successful.

**Table 1:** Numbers of paired-end raw reads and resultant numbers of trimmed reads per sample.

Family	Specie	Specie ID	Raw reads (PE)	Trimmed reads (PE)
Phyllostomidae	*Artibeus jamaicensis*	*Aj_1*	62,060,704	61,626,796
		*Aj_2*	39,814,360	39,719,296
		*Aj_3*	64,988,592	64,684,778
Mormoopidae	*Mormoops megalophylla*	*Mm_1*	45,593,364	45,518,864
		*Mm_2*	58,377,052	58,168,244
		*Mm_3*	30,645,072	30,541,808
Vespertilionidae	*Myotis keaysi*	*Mk_1*	31,849,752	31,786,368
		*Mk_2*	54,027,476	53,516,628
		*Mk_3*	60,492,802	60,371,806
Molossidae	*Nyctinomops laticaudatus*	*Nl_1*	69,645,572	69,518,612
		*Nl_2*	52,449,816	52,079,044
		*Nl_3*	63,776,122	63,563,568
Emballonuridae	*Peropteryx macrotis*	*Pm_1*	56,077,818	55,908,064
		*Pm_2*	61,404,902	61,306,544
		*Pm_3*	45,156,528	54,058,470

### De novo transcriptome assembly

Although several reference genomes and transcriptomes of bats are available, in the present study, transcripts were assembled by *de novo* instead of by genome reference mapping to decrease the possibility of losing novel or rare transcripts and species-specific sequences. *De novo* transcriptome assembly was performed with the Trinity^16^ bioinformatics tool by applying two strategies: The first strategy involved transcript reconstruction for each biological replicate; this reconstruction yielded 15 assemblies in total (three assemblies per species). For the second strategy we merged the three replicates of each species, to obtain a more complete assembly, *i.e*., one assembly per species instead of one per individual. The concatenated assemblies showed higher N50 values and higher completeness. The subsequent analysis and results presented in this publication correspond to the concatenated assembly, statistics from each assembly are presented in Table 2.

**Table 2:** *De novo assembly* Trinity statistics for each specie.

Specie ID	Total assembled bases	Total contigs	Median contig length	Average contig length
*Aj*	682,409,915	895,777	380 bp	761.81 bp
*Mm*	447,444,971	583,906	354 bp	766.30 bp
*Mk*	413,911,296	603,100	357 bp	686.31 bp
*Nl*	644,693,807	806,519	391 bp	799.35 bp
*Pm*	665,288,350	831,636	382 bp	799.98 bp

*M. keaysi* showed the lowest number of assembled bases (413.9 Mb) and a total of 603,100 trinity transcripts, these numbers are higher than those reported for the assembled transcriptomes of *Myotis ricketti*^3,17^, which included 82,000–104,000 contigs.

*A. jamaicensis* presented the highest number of assembled bases (682.4 Mb) and therefore a greater number of assembled transcripts (895,777), the number of contigs was significantly greater than in a previous study of *A. jamaicensis* that reported 214,707 contigs assembled from spleen with SOAPdenovo^12^.

According to N50 values, 50% of the assembled bases were incorporated in transcripts between 1,100 and 1,500 nucleotides in length. A preliminary assessment of *de novo* assembly quality was performed by obtaining the percentage of paired reads represented in the assembly. For *A. jamaicensis, M. megalophylla, M. keaysi, N. laticaudatus* and *P. macrotis*, the overall alignment rates were 91.82, 92.96, 93.76, 92.93 and 91.67% respectively. We interpreted these results as a first indication of a good quality assembly, based on the Trinity protocol^16^, for which it is established that 80% of read mapping should be considered indicative of good quality.

### Transcripts filtering

To reduce the number of potential spurious contigs, we filtered them based on the abundance and quality of the assembly. For abundance, we took into consideration isoforms expression level and redundancy. Expression levels were detected using the RSEM method, and only highly expressed isoforms were retained for further analysis. After this first filtering step between 76 and 81% of the assembled transcripts were conserved (Table 3).

**Table 3:** Number of filtered contigs after removal of low expressed isoforms, redundant transcripts and misassembled contigs.

Specie ID	Initial number of contigs	Highly expressed transcripts	Non-redundant transcripts	Good quality final contigs
*Aj*	895,777	700,531	685,849	674,995
*Mm*	583,906	474,259	469,620	462,172
*Mk*	603,100	462,746	456,572	260,138
*Nl*	806,519	628,231	617,036	553,868
*Pm*	831,636	646,748	634,439	450,478

Redundancy was eliminated by clustering our *de novo* assembled transcripts with highly similar contigs using CD-HIT-EST at a nucleotide identity of 95%. Fewer than 3% of the Trinity transcripts were redundant and were therefore removed (Table 3).

Regarding quality, we retrieved bad assembled contigs such as chimaeras and incomplete contigs using TransRate software. The assemblies with the highest proportion of good quality-contigs were those obtained for *A. jamaicensis* and *M. megalophylla*, which contained less than 2% of poor-quality contigs. On the other hand, *M. keaysi* contigs were depleted drastically after quality contig assessment, with 40% of poor-quality contigs that were removed for this study (Table 3). The numbers of final non-redundant transcripts considered for downstream analysis such as functional annotation, abundance quantification and differential expression analysis were 674,995 for *A. jamaicensis*, 462,172 for *M. megalophylla*, 260,138 for *M. keaysi*, 553,868 for *N. laticaudatus* and 450,478 for *P. macrotis* (Table 3).

### Assembly completeness

The results for quantitative measures for determining assembly completeness with BUSCO^18^ (Benchmarking Universal Single Copy Orthologs), showed the percentage of conserved orthologues among vertebrates, mammals and the *Laurasiatheria* superorder, represented in our assembled bat transcriptomes.

**Figure 2:**
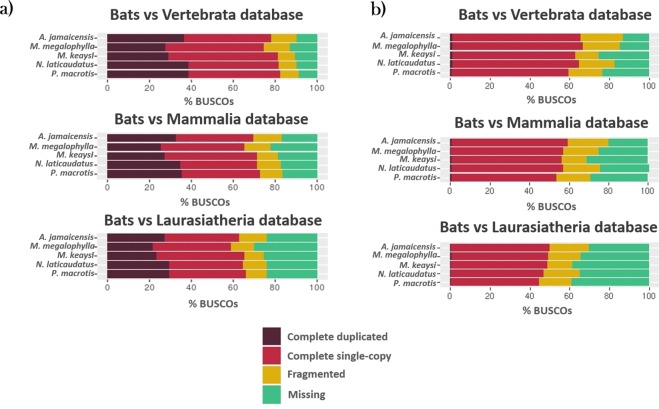
Cumulative percentage of orthologues inferred from the BUSCO search from three databases: Vertebrata, Mammalia and Laurasiatheria against non-redundant transcriptomes from five bat species (**a**), and against trimmed non-redundant sequences without weakly expressed isoforms (**b**). Complete orthologues can be either single-copy (S) or duplicated (D); incomplete orthologues are considered fragmented (F), if orthologues from databases, they are marked as missing (M).

Completeness of the non-redundant contigs containing all the isoforms resulted in a high percentage of complete vertebrate orthologues (74 to 82%), but also a significantly higher percentage of putative paralogues, *i.e*., complete genes with more than one copy (Fig. 2a). Although these results might be considered as high for genome data, for transcriptomic data they seem logical due to the presence of multiple isoforms. To probe this, we performed BUSCO analysis of the strictly filtered transcripts, excluding weakly expressed transcripts and redundant contigs; in comparing these results, we observed that the percentage of duplicates decreased markedly (Fig. 2b). Of the 2,586 orthologues searched in the BUSCO set of vertebrates, between 60 and 65% were recovered completely; of the latter, less than 1.2% were putative paralogues, *i.e*., duplicates)^18^. Of the 4,104 single-copy orthologues in mammals, 56 to 59% were complete, and 12 to 19% were partially recovered or fragmented. Finally, approximately 49% of the 6,253 laurasiatherian orthologues were complete, and 12 to 19% were fragmented (Fig. 2b). The observed recovery of more than 60 percentage of the complete single-copy orthologues from vertebrates and 50% of those from the mammalian and laurasiatherian databases is indicative of good coverage and of high recovery of conserved orthologues for the five generated non-redundant transcripts.

These results lead us to the conclusion that additional filtering steps such as debug redundancy and weakly expressed isoforms are required to increase the percent recovery of single-copy orthologues percentage and reduce the presence of putative paralogues in the assembly if required.

By comparing the completeness of our assemblies against the published transcriptomes^10,11,17,19^ of other bat species, we found that we were able to recover a higher percentage of complete vertebrate orthologues except in the case of the *Rousettus aegyptiacus*^10^ transcriptome, which presented 93% of complete orthologues. *Myotis ricketti*^17^ was the transcriptome with the lowest percentage of recovery, with only 51%, while in the case of the *Myotis keaysi* transcriptome we were able to recover 63% of the vertebrate orthologues, suggesting that the transcriptome generated in this study can be considered as a good quality reference for *Myotis* genus. For mammals and laurasiatherian orthologues, the characteristics of the database were similar (Supplementary Table S1).

### Coding regions identification and functional annotation

**Figure 3:**
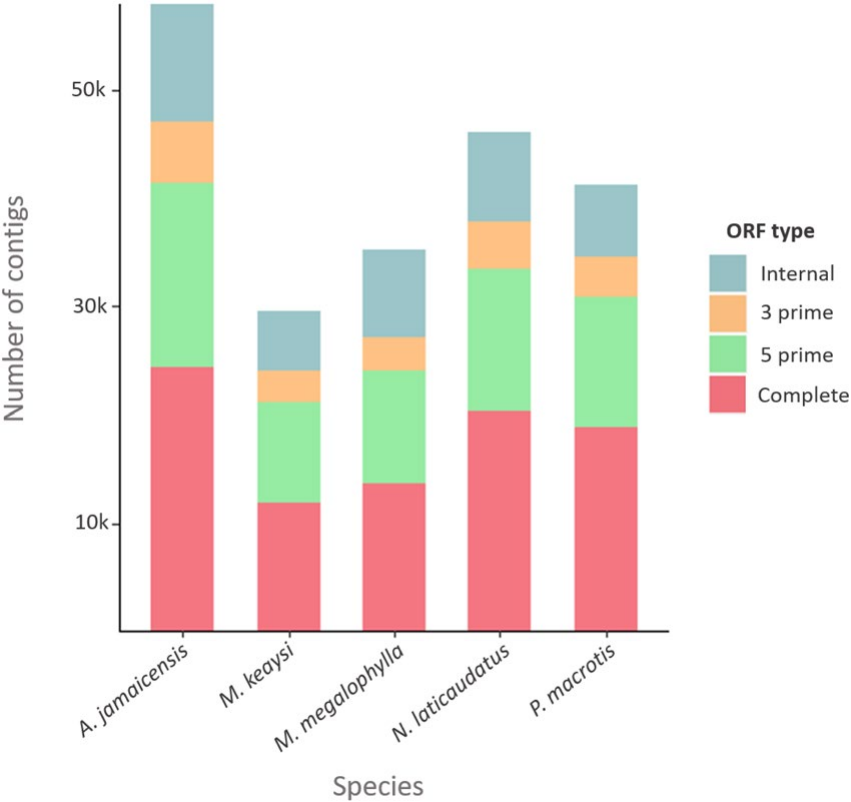
Number of transcripts with open reading frames in each species identified using the TransDecoder pipeline. “Complete ORF” refers to sequences in which the first codon and the stop codon are present. “5 prime” refers to sequences that contain the start codon but lack the stop codon; “3 primer” refers to partial ORF sequences that lack the start codon. Finally, “internal ORF” refers to sequences that lack both the start and stop codons.

**Figure 4:**
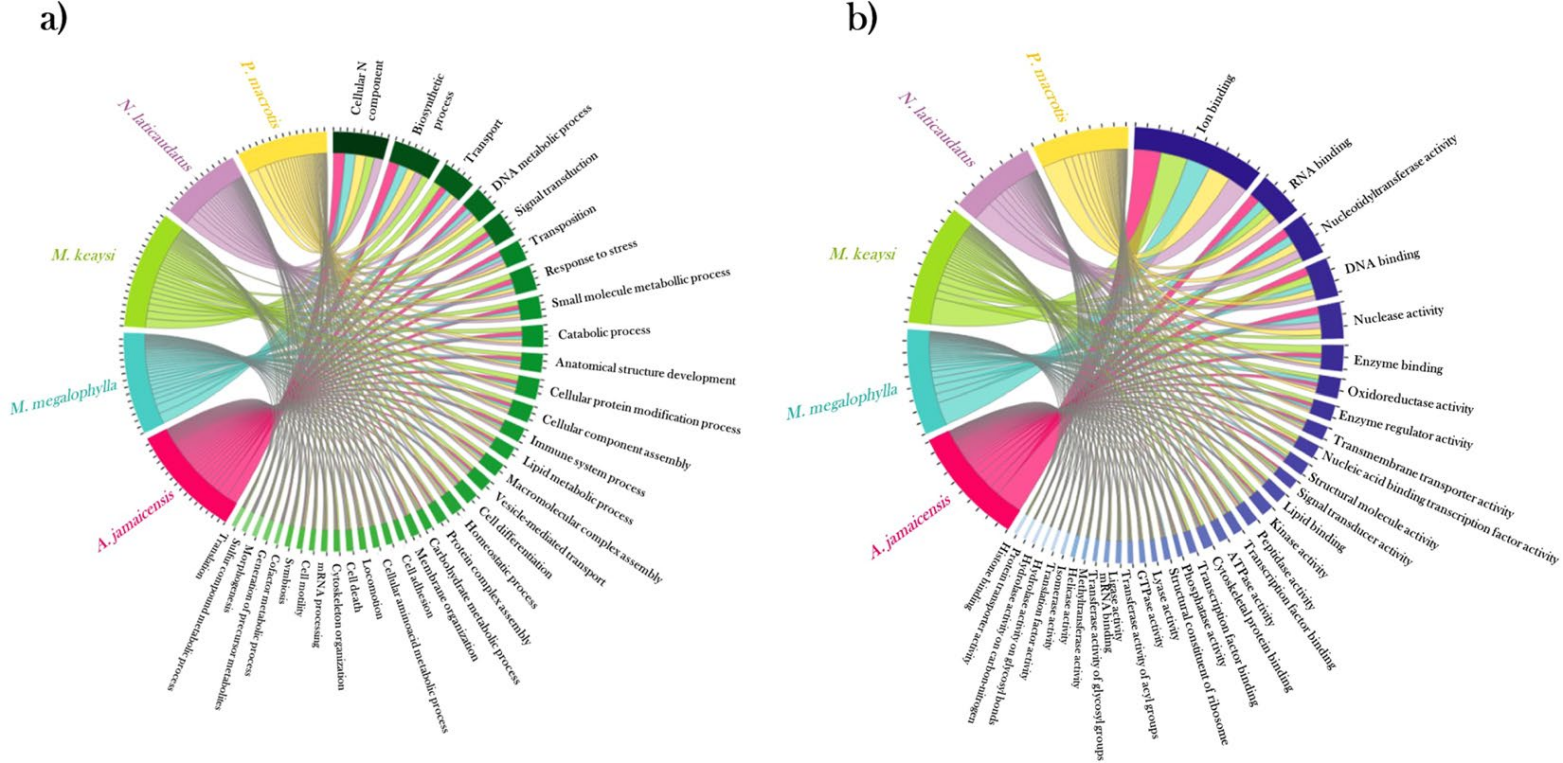
Graphic representation of Gene Ontology Abundance., Each specie is represented by a specific colour: *A. jamaicensis* is shown in magenta, *M. megalophylla* in blue, *M. keaysi* in green, *N. laticaudatus* in purple and *P. macrotis* in yellow. (**a**) Chord graph of enriched gene ontology terms for Molecular Function. (**b**) Chord graph for GO terms related to Biological Process. (**c**) GO terms for Cellular Component category.

Coding domain sequences (cds) and annotation were identified with TransDecoder for each species after two filtering steps. We first selected the single best open reading frame (ORF) per transcript, then, only transcripts more than 200 bp in length were retained. The total number of protein coding transcripts among the final non-redundant transcripts was 57,919 for *A.jamaicensis*, 35,289 for *M. megalophylla*, 29,461 for *M. keaysi*, 46,116 for *N. laticaudatus* and 41,152 for *P. macrotis*. Between 38 and 45% of the coding sequences were complete for all five species, approximately 30% were 5′ partial, 9% were 3′ partial, and 15 to 23% were internal (Fig. 3). Protein-coding transcripts were compared against the Uniprot reference database using a BLAST search with an e-value cut-off of 10^−3^. For the five species ~60% of the coding transcripts matched with this search criteria, and between 80% and 85% of these showed full-length or nearly full-length recovery, respectively. Following the Trinotate script and GOseq pipeline, Gene Ontology terms were assigned to the assembled transcriptomes. GOseq results are classified in three categories: molecular function and biological process and cellular component. The GO terms annotated within bats transcriptomes are represented in Fig. 4.

### Homology and Orthology prediction

The percentage of homology between the assembled cds transcripts against a bat-specific protein database was obtained with a BLASTp search of pairwise comparisons. The protein database was built from refseq transcriptomes of *Eptesicus fuscus, Hiposideros armiger*, *Myotis lucifugus, Pteropus alecto*, *Pteropus vampyrus* and *Rousettus aegyptiacus*, downloaded from the NCBI database.

**Figure 5:**
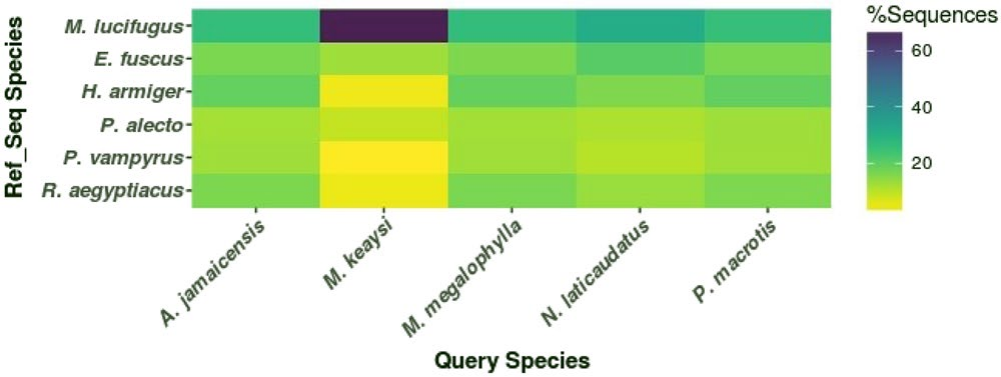
Heatmap representation of the percentage similarity between assembled transcripts and refseq sequences from six bat species (*R. aegyptiacus, P. vamyrus, P. alecto, M. lucifugus, H. armiger* and *E. fuscus*), using a BLASTp e-value cut-off < 1 × 10–5. Darker colours indicate higher percentage of similarity.

*Myotis lucifugus* presented the highest percentage of homology against all query species; as expected, the species with greatest homology was *Myotis keaysi* with 68%, whereas *A. jamaicensis, M. megalophylla, N. laticaudatus* and *P. macrotis* displayed only 25–31% homology (Fig. 5). In contrast, the *de novo* assembled transcriptomes had lower percentages of homologous transcripts (<18%) against the Yinpterochiroptera database, including *Hipposideros armiger* (F. Rhinolophidae), which was formerly classified within the suborder Microchiroptera; according to the recently proposed molecular phylogeny^1,3^, *H. armiger* was reclassified within the suborder Yinpterochiroptera along with the family Pteropodidae.

**Figure 6:**
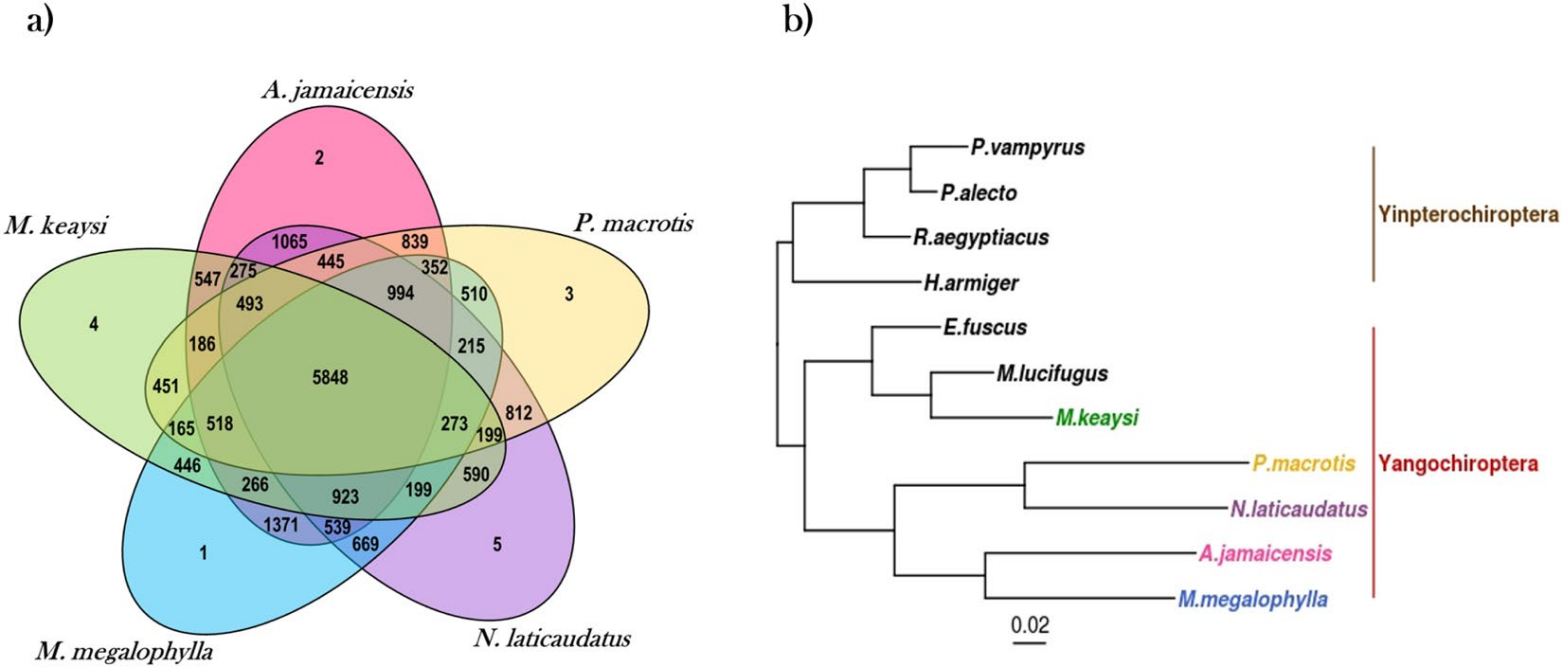
(**a**) Venn diagram representing the number of species-specific and overlapping protein orthogroups between the five bat transcriptome assemblies. The number of orthogroups were identified with Orthofinder. (**b**) Species rooted tree based in single copy orthologues, generated with Orthofinder.

Detection of putative orthologues and orthology grouping of proteins was performed with Orthofinder^20^ for the five transcriptomes using the BLAST all-v-all algorithm. A total of 209,937 transcripts were assigned to 19,205 orthogroups. An orthogroup is defined as a set of genes descended from a single gene of the last common ancestor within species groups^20^. Among the five species, 5,848 (36.4%) of the orthogroups were shared (Fig. 6a); this will be discussed further in the section on transcript abundance. A total of 72 genes were classified as species-specific, these were grouped in 15 inferred orthogroups, of which only 10 were annotated. Three orthogroups classified as species-specific from *M. keaysi* caught our special attention because these groups, according to our functional annotation, corresponded to the retroviral genes GAG, POL and ENV, respectively. The Gag p24 gene protein forms the inner protein layer of the nucleocapsid during virus replication, specifically during the assembly, maturation and infection stages of retroviruses. These findings might be a first indication that an active viral cycle was occurring at the moment of capture of the *M. keaysi* individuals used in our study. However, it is retroviral genetic material can be inserted into the host genome (endogenous retroviruses), and despite the assumption that these endo-retroviruses are not functional, in some cases they have been shown to be expressed. Further research is needed to determine whether the observed expression is due to an exogenous retrovirus or to a functional endogenous retrovirus.

In addition to detecting orthologues, Orthofinder infers a species tree based on single-copy orthogroups^20,21^. The tree was rooted with Yinpterochiroptera species as an outgroup (Fig. 6b). In the orthologues phylogram, a clear separation between the suborders Yinpterochiroptera and Yangochiroptera can be observed. As expected, the query species were classified within Yangochiroptera; *Myotis keaysi* constitutes a monophyletic group with *Eptesicus fuscus* and *Myotis lucifugus*, and all three belong to the family Vespertillionidae (Fig. 6b). The four remaining species constituted one clade with two clear divisions. *Artibeus jamaicensis* (family Phyllostomidae) and *Mormoops megalophylla* (family Mormoopidae) formed the first clade, consistent with recent molecular classification that groups them within the superfamily Noctilionoidea^1,3^. However, this was not the case for *Peropteryx macrotis* (family Emballonuridae) and *Nyctinomops laticaudatus* (family Molossidae); this clade is inconsistent with recent molecular phylogeny^1^ but agrees with two different phylogenetic reconstructions, one that was inferred from the sequences of the cytochrome B (cytb) molecular marker^3^^,22^ and another that was inferred from transcriptomic data^18^. It must be emphasized that this phylogram was not constructed under an evolutionary model but is only based on the substitution rates of single-copy orthologues.

### Transcript Abundance and Differential Expression Analysis

For estimation of the level of expression in each species, we first performed transcript quantification for each biological replicate and then used the ExN50 statistic to retrieve expression levels of the isoform level in each species. To reduce the number of comparisons, we focused only on transcripts that were shared among the five species.

These values were obtained from the ExN50 statistics calculated from theTMM matrix. For *A. jamaicensis*, the orthologous transcript with the highest expression values corresponded to the gene ALDOB which is expressed in the liver and encodes the aldolase B enzyme, a member of the glycolytic protein family (Pfam PFF00274). The fructose-biphosphate aldolase type B glycolytic enzyme catalyses the condensation of fructose 1-6-bisphosphate or fructose-1-phosphate^22,23^; the high level of expression of this enzyme in *A. jamaicensis* is clearly correlated with the species’ feeding habits as a frugivorous bat, as fructose when absorbed is metabolized in the liver by ALDOB, producing substrate for fatty acid synthesis, all this process takes place in the liver^24^. On the other hand, for the other four insectivorous bats, the most highly expressed transcripts were annotated as serum albumin protein, which is the main component of blood plasma and is related to fatty acid binding, metabolites, hormones and bilirubin. This protein was also highly expressed in *A. jamaicensis*. Other transcripts that were highly expressed in the five species corresponded to the apolipoprotein E gene (APOE). APOE is a plasma protein that is secreted primarily in hepatic tissues with a role in lipid transportation, and is also related to innate and adaptative immune response^23,25^.

**Figure 7:**
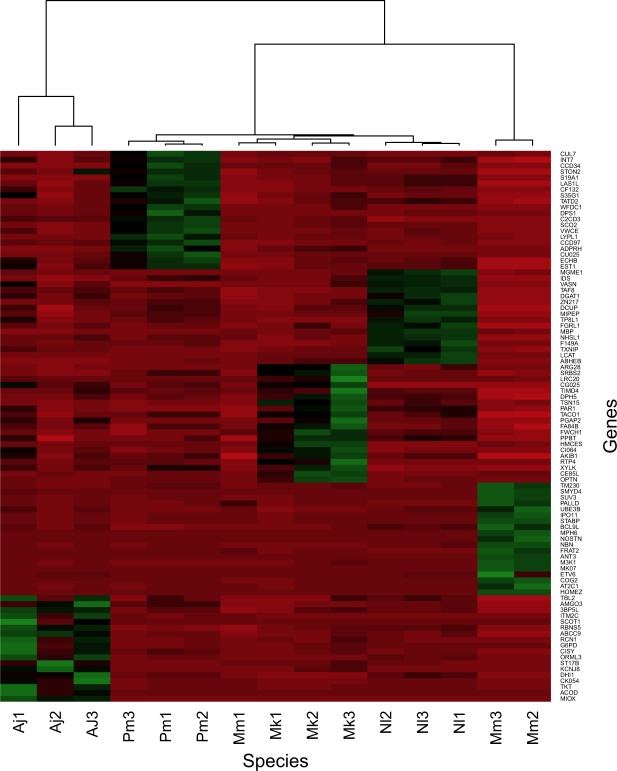
Heatmap diagram showing Differential Expressed genes (*p* < *0.01*) in liver transcriptome of five bat species. Heatmap clustering is based on transcripts abundance in the three biological replicates per specie. Up-regulated genes are represented in green and down-regulated genes are colored in red.

Now then, making a comparison between transcripts of different species with high evolutionary distances, is challenging for three main factors: (1) because we are studying wild, non-model organisms it is difficult to establish a control group that can be used to perform traditional differential expression analysis; (2) all individuals were captured under aleatory circumstances, making it impossible to know their feeding and metabolic status at the time of capture and making a comparison related to liver metabolic degradation of nutriments is not possible; (3) the lack of reference genomes for the studied species is an important obstacle to conducting a proper and reliable comparison of cross-species transcripts expression, due to the potential loss of transcript information. The ultimate (but not available) solution would be the comparison of a reference genome of at least a closely related species of each target study object; herein lies the importance of generating new available genomic and transcriptomic resources for non-model species such as bats. Therefore, to ensure that our data are as uniform as possible, we proposed, as an alternative solution, to conduct the DE analysis using only in the orthologous transcripts that were shared among the five species (Fig. 7). We retrieved 3,052 single-copy orthologues from the output of Orthofinder analysis; of these, only approximately 1,000 genes were considered differentially expressed according to the four statistical methods employed (EdgeR, DeSeq, DEseq. 2 and NOIseq). Volcano plots of each of the ten comparisons can be found in Supplementary Fig. S2. A total of 27 genes were up regulated in *Artibeus jamaicensis* against the other four species (Supplementary Table S3). Transcript that encodes for Myo-inositol oxygenase enzyme (MIOX) was the one with higher level of expression among the *A. jamaicensis* single-copy orthologues and was significantly downregulated (*p* < *0.01)* in the other four species (Fig. 7). MIOX enzyme is involved the first step of myo-inositol (MI) degradation into D-glucuronate, which is essential for the pentose phosphate cycle^26^. Upregulation of MIOX might be related to *A. jamaicensis* diet, as MI has been found in high concentrations in fruits and seeds^26,27^. In humans this enzyme is restricted to kidney expression^22^, contrary as expected, transcript abundance in the three biological replicates of *A. jamaicensis* indicates an overexpression of a MIOX like transcript in liver, further research has to be done to confirm that liver MIOX expression actually occurs in other frugivorous bat species.

Glucose-6-phoshphate dehydrogenase (G6PD) enzyme was also upregulated in *A. jamaicensis* and downregulated in the studied insectivorous species. It has been found that G6PD upregulation is related with insulin resistance metabolic syndrome^28^, which has been previously reported in organisms with high fructose levels of consumption^29^. However, more studies need to be done for probe insulin resistance in frugivorous bats as a result of an evolutionary adaptation related to feeding habits.

The biological process Gene Ontology terms involved within the upregulated genes in *A. jamaicensis* according to GO enrichment analysis were metabolic process (GO:008152), regulation of phosphate metabolic process (GO:0019220) and immune system (GO: 0071840), among others. For molecular function catalytic activities such as transferase activity and oxidoreductase activity were involved in the upregulated transcripts. The overrepresented pathways corresponded to the pentose phosphate pathway (P02762) and pyruvate metabolism (P02772). Most of the upregulated transcripts in *A. jamaicensis* are correlated with species feeding habits, in Fig. 7 it is appreciated how *Artibeus* replicates are grouped in an independent cluster separated from the insectivorous species.

Comparisons between insectivorous species are not discuss here, as species-specific upregulated single copy orthologues were not correlated with feeding habits or another specie-specific biological behaviour, other than expected liver metabolism process. A complete table of up-regulated genes and respective annotation for each insectivorous species can be consulted in Supplementary Tables S4–S7.

## Conclusions

In this study, we generated whole transcriptomes from the liver tissue of five species of tropical bats classified into five different families: *A. jamaicensis* (F. Phyllostomidae), *Mormoops megalophylla* (F. Mormoopidae), *Myotis keaysi* (F. Vespertilionidae), *Nyctinomops laticaudatus* (F. Molossidae) and *Peropteryx macrotis* (F. Emballonuridae). Also, a comparative analysis was performed to obtain overexpressed genes of each species, this comparison was performed exclusively on single-copy orthologues shared across the five species, with this cross-species differential gene expression analysis we were able to identify species-specific upregulated genes related with dietary habits in bats, however this analysis was restricted to shared orthologous transcripts due to the lack of a closest reference genome or transcriptome database. Here relies the importance of generating new genomic database for non-model species, especially for wild species with high ecological and evolutionary importance such as bats.

## Methods

### Ethic statement

Living individuals were collected under research permits issued by the Undersecretariat of Management for Environmental Protection of the Wildlife National Leadership (Subsecretaría de Gestión Para la Protección Ambiental de la Dirección Nacional de Vida Silvestre) (with permission number SGPA/DGVS/12598/15).

Handling and sacrifice methods applied to the specimens were approved at the document CB-CCBA-I-2017-006, which was issued by the Bioethics Committee of the Faculty of Veterinary Medicine and Zootechnics at Autonomous National University of Yucatan (Universidad Nacional Autónoma de Yucatán, UADY).

### Sampling collection

In this study we collected three biological replicates from each of five bat species belonging to five families (Table 1). Bats were collected from various locations in Yucatan State in south-eastern Mexico (Fig. 1). *A. jamaicensis, M. megalophylla* and *N. laticaudatus* were collected at Calcehtok cave; free-ranging bats were captured using two mist-nets located at the cave entrance. *M. keaysi* individuals were captured inside Hoctún cave using mist-nets, and *P. macrotis* were collected in their roosting at Hobonil cave using a sweep net.

The specimens were transported alive to the Arbovirology Laboratory at the University of Yucatan (Universidad Autónoma de Yucatán, UADY). There, they were sacrificed by lethal cardiac puncture and dissected according to the guidelines and regulations stated in the document CB-CCBA-I-2017-006 referenced in the Ethics Statement section. Organs and tissues were stored in RNAlater buffer and frozen at −20 °C for later use.

### RNA extraction and transcriptomic sequencing

Total RNA was extracted from hepatic tissue using an RNAeasy extraction kit from QIAGEN®^30^ with a DNAse cleaning step according to the manufacturer’s instructions. RNA quality was measured using Nanodrop, Qubit 2.0 fluorometer and Bioanalyzer 2100 instruments.

Fifteen libraries (one per individual) were constructed from 1.0 *µl* of total RNA using the KAPA Stranded RNA-Seq kit with RiboErase from KapaBiosystems®. Ribosomal RNA was removed by depletion by DNA primer hybridization followed by treatment with RNAse and DNAse according to the manufacturer’s instructions. The fragmentation cycle was adjusted to 10 cycles of amplification at 94 °C for 5 minutes to obtain fragments between 100 and 2100 bp in length. Each library was enriched with Illumina® TruSeq Index Adapters (250 nM) for multiplex sequencing.

Library quality was evaluated using a Qubit 2.0 fluorometer and an Agilent Bioanalyzer 2100; good quality libraries were paired-end (PE) sequenced on an Illumina HiSeq. 4000 at the Center for Genomics Services of the University of California, Berkeley.

### De novo transcriptome assembly

The raw read quality of each paired-end library was examined using the bioinformatics tool FastQC v 0.11.5^31^. Adapters were removed using cutadapt v. 1.12^15^ for paired-end reads (R1 and R2), in addition poly A/T tails, ambiguities (N), sites with PHRED scores lower than 20 and reads below 30 bp in length were removed. For this purpose, the following parameters were used: “-a <*adapter sequence*> - A<*adapter sequence>*- o<*output_read_1.fastq*> -p<*output_read_2.fastq*> -q 20 -b “A{101}” -B “T{101}” -trim-n –minimum-length 30 -o<*output_read_1.fastq*> -p<*output_read_2.fastq*>”; for adapter trimming we added an “A” at the beginning of each adapter sequence. The edited reads were re-examined on FastQC v 0.11.5^31^ to verify their final quality.

Transcriptome *de novo* assembly was performed for each species using Trinity v.2.4.0^16^ with previous normalization of the edited reads. The following parameters were used: “–seqType fq–SS_lib_type RF–left<input_file>–right<input_file> –CPU 62–max_memory 400 G”. Assembly statistics were computed using the script TrinityStats.pl contained in the Trinity^16^ package. The proportion of reads mapped to the assembly was assessed with Bowtie2^32^.

### Assembly filtering

To reduce the probability of obtaining of spurious transcripts and attenuate transcript redundancy, the contigs were filtered using three methods: First, weakly expressed isoforms were removed based on their expression values^16^. TPM values were obtained by the RSEM^33^ method using Trinity script *aling_and_estimate_abundance.pl*; then, weakly expressed isoforms were removed using the Trinity script *filter_low_expr_transcripts.pl* with “–highest_iso_only” parameter. Second, a set of non-redundant representative transcripts was generated using the CD-Hit^34^ package with an identity threshold of 95%. The parameters used were “cd-hit-est -c 0.95 -n 10 -M 60000 -T 8”.

Finally, bad contigs, *i.e*., misassembled or incomplete contigs, were filtered out from non-redundant assemblies based on read mapping metrics. For this purpose, we used the bioinformatics tool TransRate^35^, which is designed for analysis of the quality of de novo transcriptome assemblies. Downstream analysis was performed on the final filtered contigs.

### Assembly completeness

Transcriptome completeness was assessed using the bioinformatics tool BUSCO *v.3*^18^ *(Benchmarking Universal Single-Copy Orthologs)* to obtain the percentage of single-copy orthologues represented in three datasets: Vertebrate odb9, Mammalia odb9 and the superorder Laurasiatheria odb9. Furthermore, to compare the completeness of our assemblies against other bat species, we performed BUSCO analysis of the transcriptomes of five other species downloaded from the NCBI database: *Cynopterus sphinx*^17^, (BioPoject:PRJNA198831), *Desmodus rotundus*^19^ (BioProject: PRJNA178123), *Myotis ricketti*^17^ (BioPoject:PRJNA222414), *Rhinolophus ferrumequinum*^11^ (BioProject: PRJNA238288) and *Rousettus aegyptiacus*^10^ (BioProject: PRJNA300284). Plotting of the BUSCO results w*as* performed using the ggplot2^36^ package contained in RStudio^37^.

### Homology Search and Gene Orthology Prediction

Analysis of homology between *de novo* assembled transcripts and the bat refseq database was performed by pairwise comparison using BLAST to provide full-length transcript analysis. Bat databases were constructed with the option -*makeblastdb* included in the BLAST software. For the BLASTn and BLASTp analysis, we built a database of the refseq genome sequences and coding DNA sequences from seven species: *Rousettus aegyptiacus* (BioProject: PRJNA309421), *Pteropus vampyrus* (BioProject: PRJNA275879), *Pteropus alecto* (BioProject: PRJNA232518), *Eptesicus fuscus* (BioProject: PRJNA232522), *Hipposideros armiger* (BioProject: PRJNA357596) and *Myotis lucifugus* (BioProject: PRJNA208947).

Orthologous groups (orthogroups) of protein sequences amongst the five species were identified with the Orthofinder v.2.1.2^20^ bioinformatics tool, using default parameters. Using reciprocal best-hits by the BLAST all-v-all algorithm, Orthofinder determined the number of shared putative orthologues between the five species as well as species-specific transcripts. Graphic representation for Orthofinder output was performed using the VennDiagram^38^ package v. 1.6.20 contained in RStudio^37^.

A gene tree was constructed using with with Orthofinder v.2.1.2^20^, based on the STAG (Species Tree Inference form All Genes) method. Gene tree inference was performed by calling default parameters, which use the MAFFT^39^ program to generate the alignment and FastTree^40^ for tree inference. Six reference bat transcriptomes were incorporated into the previously computed analysis to make it possible to perform extra BLAST all-v-all searches and recalculation of orthogroups^20^. The transcriptomes used for gene tree inference were those of *Rousettus aegyptiacus* (BioProject:PRJNA309421), *Pteropus vampyrus* (BioProject: PRJNA275879)*, Pteropus alecto* (BioProject: PRJNA232518), *Eptesicus fuscus* (BioProject: PRJNA232522), *Hipposideros armiger* (BioProject: PRJNA357596) and *Myotis lucifugus* (BioProject: PRJNA208947). The phylogram was manually rooted with dendroscope v.3.5.9^41^. Tree visualization and annotation were performed using the R Bioconductor package ggtree^42^.

### Functional Annotation

Candidate coding regions with a minimum cut-off of 200 amino acids and open reading frames (OFRs) were predicted with TransDecoder^16^ pipeline. Once the candidate peptides we obtained, we performed homology searches against known proteins using BLASTp using UniProtKB/Swiss-Prot database and against common protein domains using the Pfam^43^ database. The BLAST and Pfam search outputs were integrated into coding regions prediction. Functional annotation was conducted using the Trinotate^16^ utility (http://trinotate.sourceforge.net/), in which homology search was performed with BLASTx, whilst for predicted coding region homology search we used BLASTp. Additionally, protein domains were identified with HMMER v.3.1^44^ against the Pfam database. Signal peptide predictions was performed using signalP v.4^45^. Transmembrane regions were predicted using the tmHMM v.2^46^ server and ribosomal RNA genes were detected with RNAMMER v.1.2^47^. Annotation outputs were loaded into a Trinotate SQLite Database.

### Transcript Abundance and Differential Expression Analysis

To quantify transcript abundance we applied the alignment-based methods contained in the Trinity^16^ package, by mapping the reads of each biological replicate against the respective assembled transcriptome. This was obtained with the align_and_estimate_abundance Perl script. In this analysis, we used RSEM^33^ as the abundance estimation method and chose bowtie2^48^ for the alignment. When the transcript abundance for each biological replicate had been obtained, we built a Gene Expression Matrix using the abundance_estimates_to_matrix.pl script to generate a normalized expression values matrix that was used to obtain the expression level of each transcript by ExN50 analysis.

Differential expression analysis (DE) was performed only on transcripts for which orthologues were present in the five species; such sequences were obtained from Orthofinder analysis. For DE, quantification of orthologous transcripts was performed using the Salmon^49^ tool, a quasi-map index was built with each species assembled transcriptome, using a k value of 29, as the shortest length of the reads was 30 bp. As with RSEM, we aligned each biological replicate to its transcriptome. Each quantification file was edited by replacing the transcript ID generated by Trinity, for its respective Single Gene Orthogroup name. This was necessary because each assembly was generated independently and without any reference, therefore the Trinity ID headers were assigned randomly to each species. By assigning the orthologue ID we propose that a more accurate differential expression analysis in non-related species can be performed.

The edited quantification files generated by Salmon software were imported to R using the tximport^50^ package contained in the Bioconductor^51^ library. The counts matrix filed imported was submitted to the web tool IDEAmex^52^ (*Integrated Differential Expression Analysis MultiEXperiment*, http://zazil.ibt.unam.mx/idea) which performed differential expression analysis, taking into consideration the biological replicates with four methods: EdgeR, NOISeq, DESeq and DESeq. 2. In this analysis, we used only genes that were differentially expressed according to the four methods. Finally, GeneOntology (GO) and KEGG enrichment were obtained from the PANTHER web tool, using *Sus scrofa* as a reference.

## Supplementary information

Combined_Supplementary

## Data Availability

The datasets generated at the current research, were deposited in the NCBI database under BioProjectID PRJNA490553.

## References

[1] Teeling EC (2005). A Molecular Phylogeny for Bats Illuminates Biogeography and the Fossil Record. Science..

[2] Kunz TH, de Torrez EB, Bauer D, Lobova T, Fleming TH (2011). Ecosystem services provided by bats. Ann. N. Y. Acad. Sci..

[3] Lei M, Dong D (2016). Phylogenomic analyses of bat subordinal relationships based on transcriptome data. Sci. Rep..

[4] Teeling EC (2000). Molecular evidence regarding the origin of echolocation and flight in bats. Nature.

[5] Shen Y-Y (2010). Adaptive evolution of energy metabolism genes and the origin of flight in bats. Proc. Natl. Acad. Sci..

[6] Zhang G (2013). Comparative Analysis of Bat Genomes. Science..

[7] Papenfuss Anthony T, Baker Michelle L, Feng Zhi-Ping, Tachedjian Mary, Crameri Gary, Cowled Chris, Ng Justin, Janardhana Vijaya, Field Hume E, Wang Lin-Fa (2012). The immune gene repertoire of an important viral reservoir, the Australian black flying fox. BMC Genomics.

[8] Ng JHJ (2016). Evolution and comparative analysis of the bat MHC-I region. Nat. Publ. Gr..

[9] Wang, L. F. & Cowled, C. Bats and Viruses: A New Frontier of Emerging Infectious Diseases. Bats and Viruses: A New Frontier of Emerging Infectious Diseases (2015).

[10] Lee, A. K. *et al*. De novo transcriptome reconstruction and annotation of the Egyptian rousette bat. *BMC Genomics* **16** (2015). 10.1186/s12864-015-2124-x PMC4672546 26643810

[11] Lei, M., Dong, D., Mu, S., Pan, Y. H. & Zhang, S. Comparison of brain transcriptome of the greater horseshoe bats (Rhinolophus ferrumequinum) in active and torpid episodes. *PLoS One* **9** (2014). 10.1371/journal.pone.0107746 PMC4174523 25251558

[12] Shaw TI (2012). Transcriptome Sequencing and Annotation for the Jamaican Fruit Bat (Artibeus jamaicensis). PLoS One.

[13] Huang, Z., Jebb, D. & Teeling, E. C. Blood miRNomes and transcriptomes reveal novel longevity mechanisms in the long-lived bat, Myotis myotis. *BMC Genomics* (2016). 10.1186/s12864-016-3227-8 PMC5103334 27832764

[14] Foley Nicole M., Hughes Graham M., Huang Zixia, Clarke Michael, Jebb David, Whelan Conor V., Petit Eric J., Touzalin Frédéric, Farcy Olivier, Jones Gareth, Ransome Roger D., Kacprzyk Joanna, O’Connell Mary J., Kerth Gerald, Rebelo Hugo, Rodrigues Luísa, Puechmaille Sébastien J., Teeling Emma C. (2018). Growing old, yet staying young: The role of telomeres in bats’ exceptional longevity. Science Advances.

[15] Martin M (2011). Cutadapt removes adapter sequences from high-throughput sequencing reads. EMBnet. journal.

[16] Haas BJ (2013). De novo transcript sequence reconstruction from RNA-seq using the Trinity platform for reference generation and analysis. Nat. Protoc..

[17] Dong, D., Lei, M., Liu, Y. & Zhang, S. Comparative inner ear transcriptome analysis between the Rickett’s big-footed bats (Myotis ricketti) and the greater short-nosed fruit bats (Cynopterus sphinx). *BMC Genomics* **14** (2013). 10.1186/1471-2164-14-916 PMC3879654 24365273

[18] Simão FA, Waterhouse RM, Ioannidis P, Kriventseva EV, Zdobnov EM (2015). BUSCO: Assessing genome assembly and annotation completeness with single-copy orthologs. Bioinformatics.

[19] Francischetti IMB (2013). The ‘Vampirome’: Transcriptome and proteome analysis of the principal and accessory submaxillary glands of the vampire bat Desmodus rotundus, a vector of human rabies. J. Proteomics.

[20] Emms, D. M. & Kelly, S. OrthoFinder: solving fundamental biases in whole genome comparisons dramatically improves orthogroup inference accuracy. *Genome Biol*. **16**, (2015). 10.1186/s13059-015-0721-2 PMC4531804 26243257

[21] Agnarsson, I., Zambrana-Torrelio, C. M., Flores-Saldana, N. P. & May-Collado, L. J. A time-calibrated species-level phylogeny of bats (chiroptera, Mammalia). *PLoS Curr*. (2011). 10.1371/currents.RRN1212 PMC3038382 21327164

[22] Finn, R. D. *et al*. Pfam: The protein families database. *Nucleic Acids Research* **42** (2014). 10.1093/nar/gkt1223 PMC3965110 24288371

[23] Amberger JS, Bocchini CA, Schiettecatte F, Scott AF, Hamosh A (2015). OMIM: online mendelian inheritance in man (OMIM). Nucleic Acids Res..

[24] Munnich A (1985). Dietary and hormonal regulation of aldolase B gene expression. J. Clin. Invest..

[25] Getz GS, Reardon CA (2009). Apoprotein E as a lipid transport and signaling protein in the blood, liver, and artery wall. J. Lipid Res..

[26] Croze ML, Soulage CO (2013). Potential role and therapeutic interests of myo-inositol in metabolic diseases. Biochimie.

[27] Masuda T (2003). Quantitative determination of sugars and myo-inositol in citrus fruits grown in Japan using high-performance anion-exchange chromatography. J. Nutr. Sci. Vitaminol. (Tokyo)..

[28] Park J (2006). Increase in glucose-6-phosphate dehydrogenase in adipocytes stimulates oxidative stress and inflammatory signals. Diabetes.

[29] Koo HY (2008). Dietary fructose induces a wide range of genes with distinct shift in carbohydrate and lipid metabolism in fed and fasted rat liver. Biochim. Biophys. Acta - Mol. Basis Dis..

[30] Qiagen. RNA Purification: Qiagen RNeasy Micro Handbook [2003]. Prod. Lit. Qiagen 1–76 (2003).

[31] Andrews, S. FastQC: A quality control tool for high throughput sequence data. http://www.Bioinformatics.Babraham.Ac.Uk/Projects/Fastqc/, http://www.bioinformatics.babraham.ac.uk/projects/ (2010).

[32] Langmead, B., Trapnell, C., Pop, M. & Salzberg, S. L. Bowtie: An ultrafast memory-efficient short read aligner. *Genome Biol*. **R25** (2009). 10.1186/gb-2009-10-3-r25 PMC2690996 19261174

[33] Li, B. & Dewey, C. N. RSEM: Accurate transcript quantification from RNA-seq data with or without a reference genome. in Bioinformatics: The Impact of Accurate Quantification on Proteomic and Genetic Analysis and Research 41–74 (2014). 10.1186/1471-2105-12-323 PMC3163565 21816040

[34] Li W, Godzik A (2006). Cd-hit: A fast program for clustering and comparing large sets of protein or nucleotide sequences. Bioinformatics.

[35] Smith-Unna R, Boursnell C, Patro R, Hibberd JM, Kelly S (2016). TransRate: Reference-free quality assessment of de novo transcriptome assemblies. Genome Res..

[36] Wickham, H. Ggplot 2. Elegant Graphics for Data Analysis (2009).

[37] R Studio Team. R Studio: Integrated Development for R. R Studio. (R Studio, Inc., Boston, MA, 2015).

[38] Chen, H. & Boutros, P. C. VennDiagram: A package for the generation of highly-customizable Venn and Euler diagrams in R. *BMC Bioinformatics* **12** (2011). 10.1186/1471-2105-12-35 PMC3041657 21269502

[39] Benning C (2009). MAFFT - a multiple sequence alignment program. Annual Review of Cell and Developmental Biology.

[40] Price MN, Dehal PS, Arkin AP (2009). Fasttree: Computing large minimum evolution trees with profiles instead of a distance matrix. Mol. Biol. Evol..

[41] Huson, D. H. *et al*. Dendroscope: An interactive viewer for large phylogenetic trees. *BMC Bioinformatics* **8** (2007). 10.1186/1471-2105-8-460 PMC2216043 18034891

[42] Yu, G., Smith, D. K., Zhu, H., Guan, Y. & Lam, T. T.-Y. ggtree: an R package for visualization and annotation of phylogenetic tree with different types of meta-data. *Methods Ecol. Evol*. **8**, Submitted (2017).

[43] Finn RD (2016). The Pfam protein families database: Towards a more sustainable future. Nucleic Acids Res..

[44] Finn, R. D., Clements, J. & Eddy, S. R. HMMER web server: Interactive sequence similarity searching. *Nucleic Acids Res*. **39** (2011). 10.1093/nar/gkr367 PMC3125773 21593126

[45] Petersen TN, Brunak S, Von Heijne G, Nielsen H (2011). SignalP 4.0: Discriminating signal peptides from transmembrane regions. Nature Methods.

[46] Krogh A, Larsson B, von Heijne G, Sonnhammer EL (2001). Predicting transmembrane protein topology with a hidden markov model: application to complete genomes 11 Edited by F. Cohen. J. Mol. Biol..

[47] Lagesen K (2007). RNAmmer: Consistent and rapid annotation of ribosomal RNA genes. Nucleic Acids Res..

[48] Langmead B, Salzberg SL (2012). Fast gapped-read alignment with Bowtie 2. Nat. Methods.

[49] Patro R, Duggal G, Love MI, Irizarry RA, Kingsford C (2017). Salmon provides fast and bias-aware quantification of transcript expression. Nat. Methods.

[50] Soneson C, Love MI, Robinson MD (2016). Differential analyses for RNA-seq: transcript-level estimates improve gene-level inferences. F1000 Research.

[51] Gentleman R, Carey V (2002). Bioconductor. R News.

[52] Vega-Alvarado, L. & Jimenez-Jacinto, V. IDEAmex: Integrated Diferencial Expresion Analysis Multi experiment 1–26 (2018).

