# Supplementary material for: De Novo Transcriptome Assembly and Functional Annotation in Five Species of Bats
Source: Sci Rep. 2019 Apr 17;9:6222. doi: 10.1038/s41598-019-42560-9 (PMC6470166; doi:10.1038/s41598-019-42560-9)
Supplement: Supplementary file 1 — Combined_Supplementary [file 41598_2019_42560_MOESM1_ESM.docx]

**DE NOVO TRANSCRIPTOME ASSEMBLY AND FUNCTIONAL ANNOTATION IN FIVE SPECIES OF BATS**

**Diana D Moreno-Santillán^1^, Carlos Machain-Williams^2^, Georgina Hernández-Montes^3^, and Jorge Ortega Reyes^1*^**

^1^ Escuela Nacional de Ciencias Biológicas, Instituto Politécnico Nacional, Posgrado en Ciencias Biológicas, Departamento de Zoología, Ciudad de México, México.

^2^Centro de Investigaciones Regionales Dr. Hideyo Noguchi, Universidad Autónoma de Yucatán, Laboratorio de Arbovirología. Yucatán, México.

^3^Universidad Nacional Autónoma de México, Red de Apoyo a la Investigación, Ciudad de México, México.

**Supplementary file S1**. Percentage of single copy orthologues inferred from Vertebrata, Mammalia and Laurasiatheria database against previously published bat transcriptomes assemblies downloaded from the NCBI database resource.

| BUSCO SEARCH OF BATS VS VERTEBRATA DATABASE | | | | | |
| --- | --- | --- | --- | --- | --- |
|  | *Cynopterus sphinx* | *Desmodus rotundus* | *Myotis ricketti* | *Rhinolophus ferrumequinum* | *Rousettus aegyptiacus* |
| Complete | 65,4 | 62,9 | 51,4 | 64,4 | 93,4 |
| Complete Single Copy | 41,4 | 56,6 | 38 | 52,3 | 89,3 |
| Complete Duplicated | 24 | 6,3 | 13,4 | 12,1 | 4,1 |
| Fragmented | 24,4 | 1,8 | 29,8 | 22,9 | 4,3 |
| Missing | 10,2 | 35,3 | 18,8 | 12,7 | 2,3 |
| BUSCO SEARCH OF BATS VS MAMMALIA DATABASE | | | | | |
|  | *Cynopterus sphinx* | *Desmodus rotundus* | *Myotis ricketti* | *Rhinolophus ferrumequinum* | *Rousettus aegyptiacus* |
| Complete | 57,8 | 55,2 | 42,2 | 56 | 92,5 |
| Complete Single Copy | 35,1 | 48,2 | 30,2 | 44,4 | 89,2 |
| Complete Duplicated | 22,7 | 7 | 12 | 11,6 | 3,3 |
| Fragmented | 25,1 | 1 | 28,4 | 20,9 | 4,7 |
| Missing | 17,1 | 43,8 | 29,4 | 23,1 | 2,8 |
| BUSCO SEARCH OF BATS VS LAURASIATHERIA DATABASE | | | | | |
|  | *Cynopterus sphinx* | *Desmodus rotundus* | *Myotis ricketti* | *Rhinolophus ferrumequinum* | *Rousettus aegyptiacus* |
| Complete | 48,90% | 50,5 | 35,9 | 50,5 | 87,2 |
| Complete Single Copy | 30,7 | 45,1 | 26,2 | 40 | 83,7 |
| Complete Duplicated | 18,2 | 5,4 | 9,7 | 10,5 | 3,5 |
| Fragmented | 22,7 | 1,4 | 21,5 | 16,6 | 6,5 |
| Missing | 28,4 | 48,1 | 42,6 | 32,9 | 6,3 |

**Supplementary file 2.** Volcano plots for cross-species differential expression analysis using common single-copy orthologous. DE analysis was performed with the DEseq2 bioconductor package in RStudio.


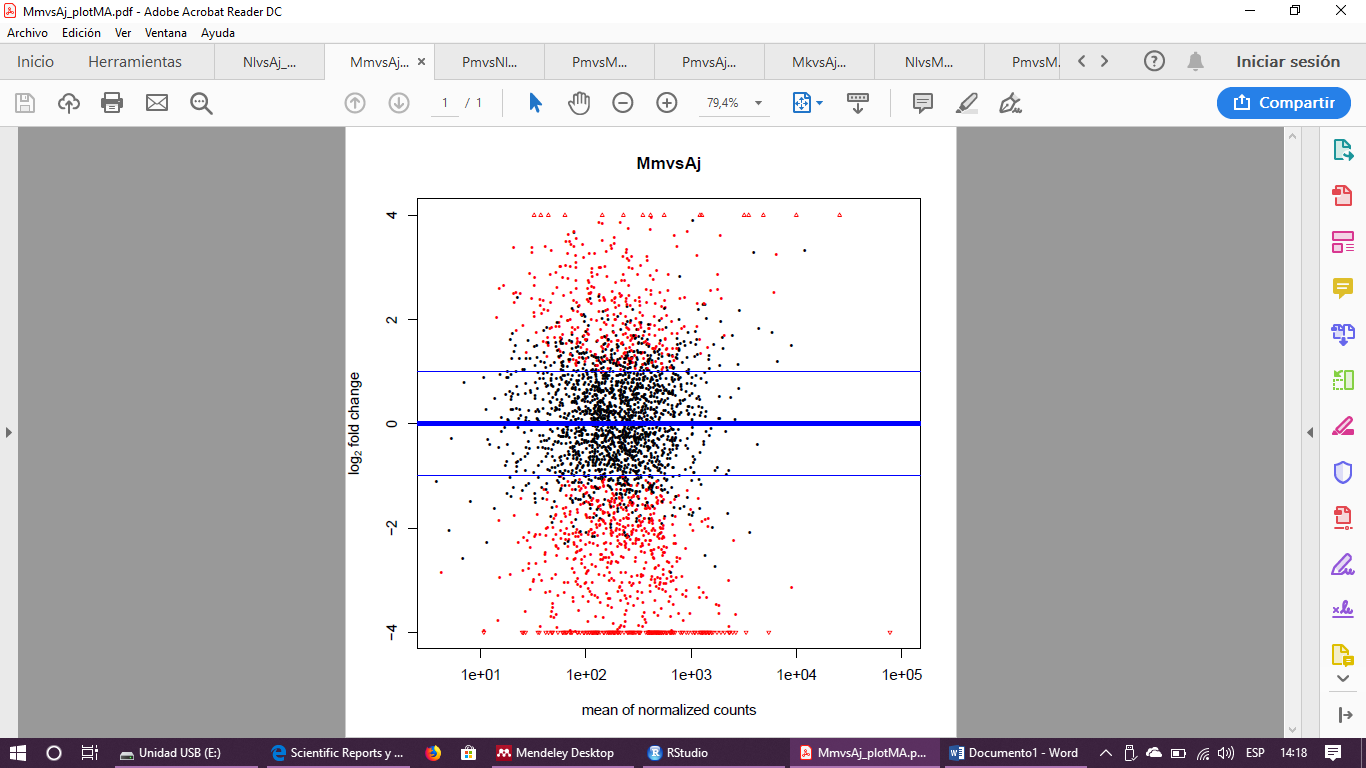


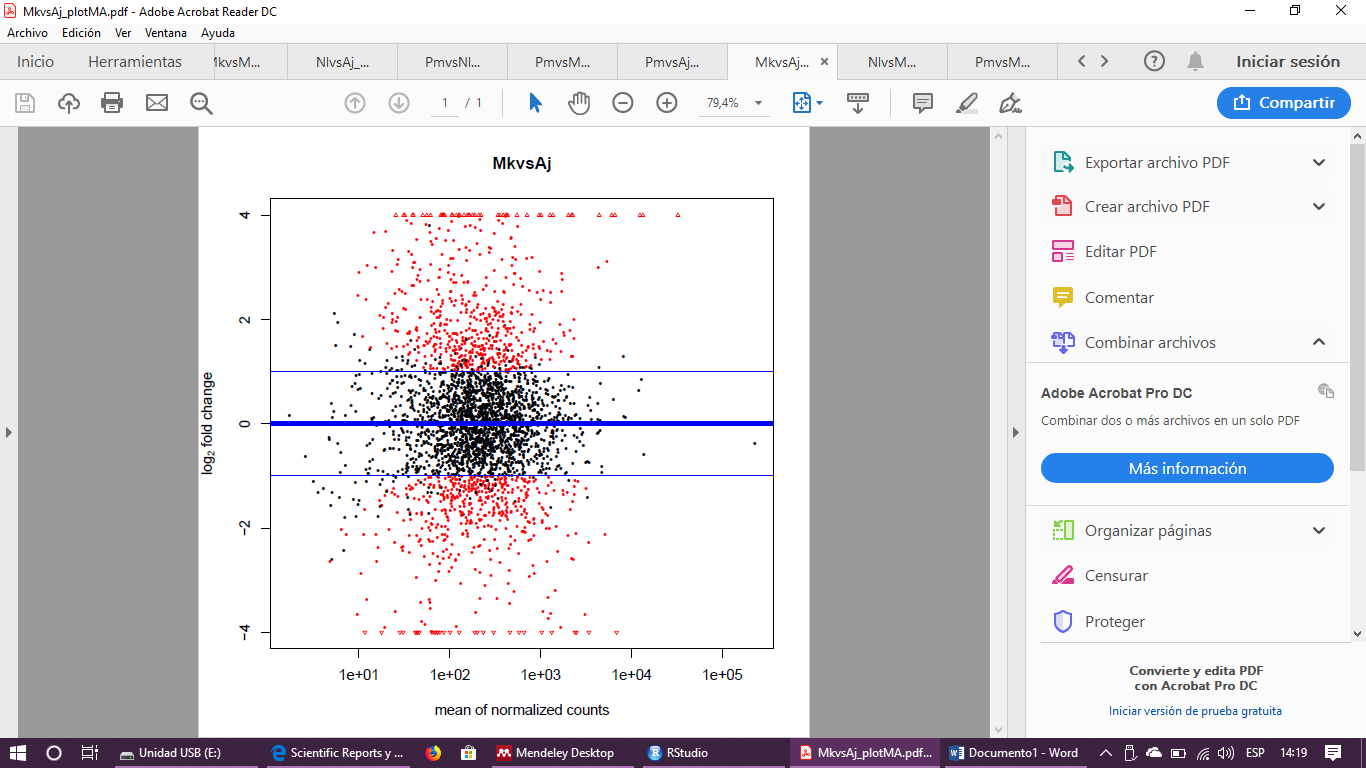


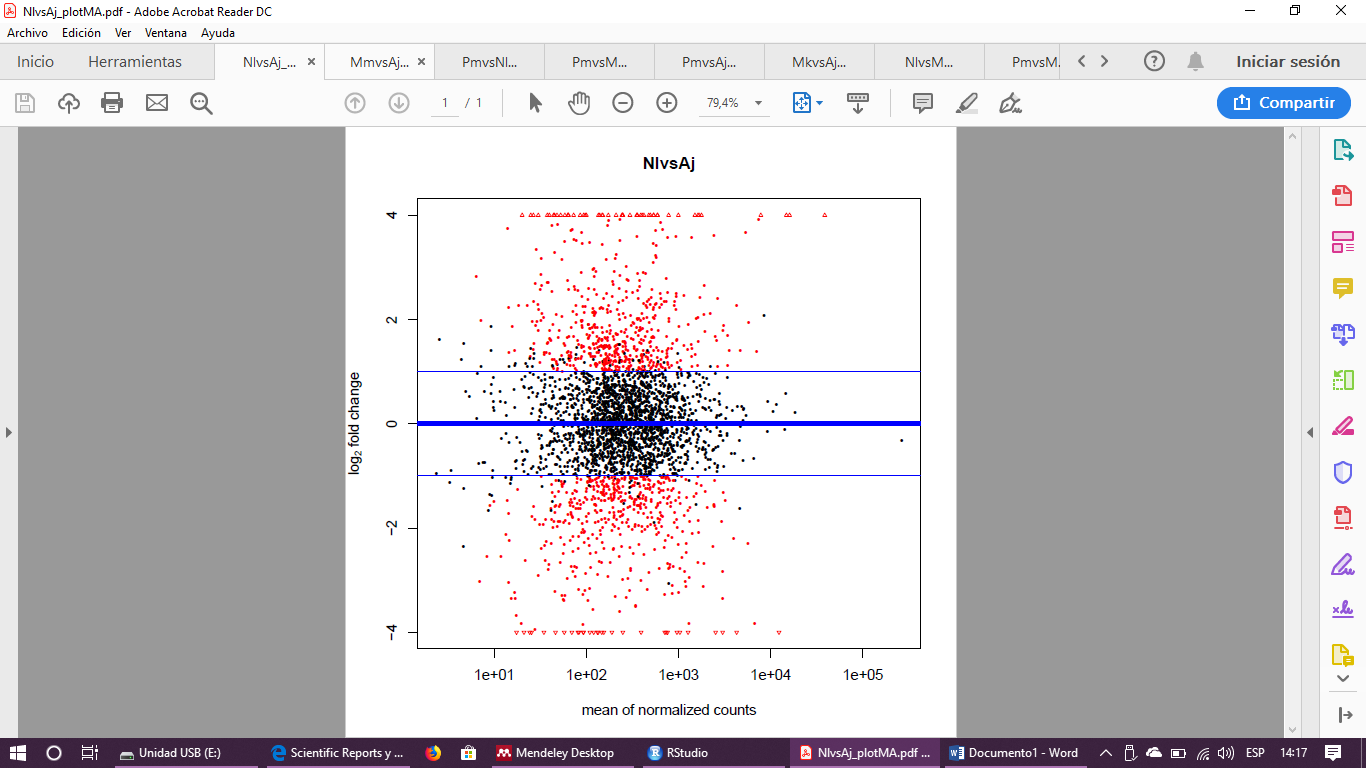


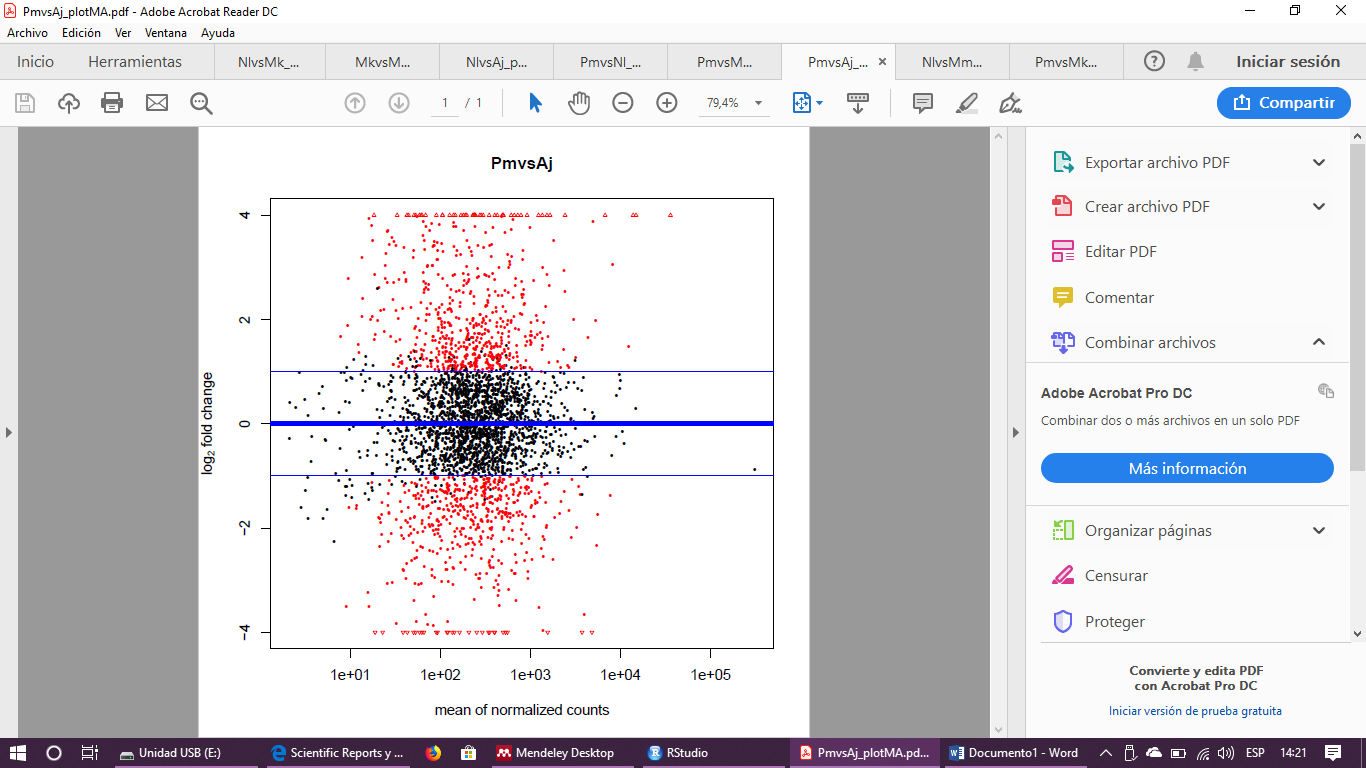


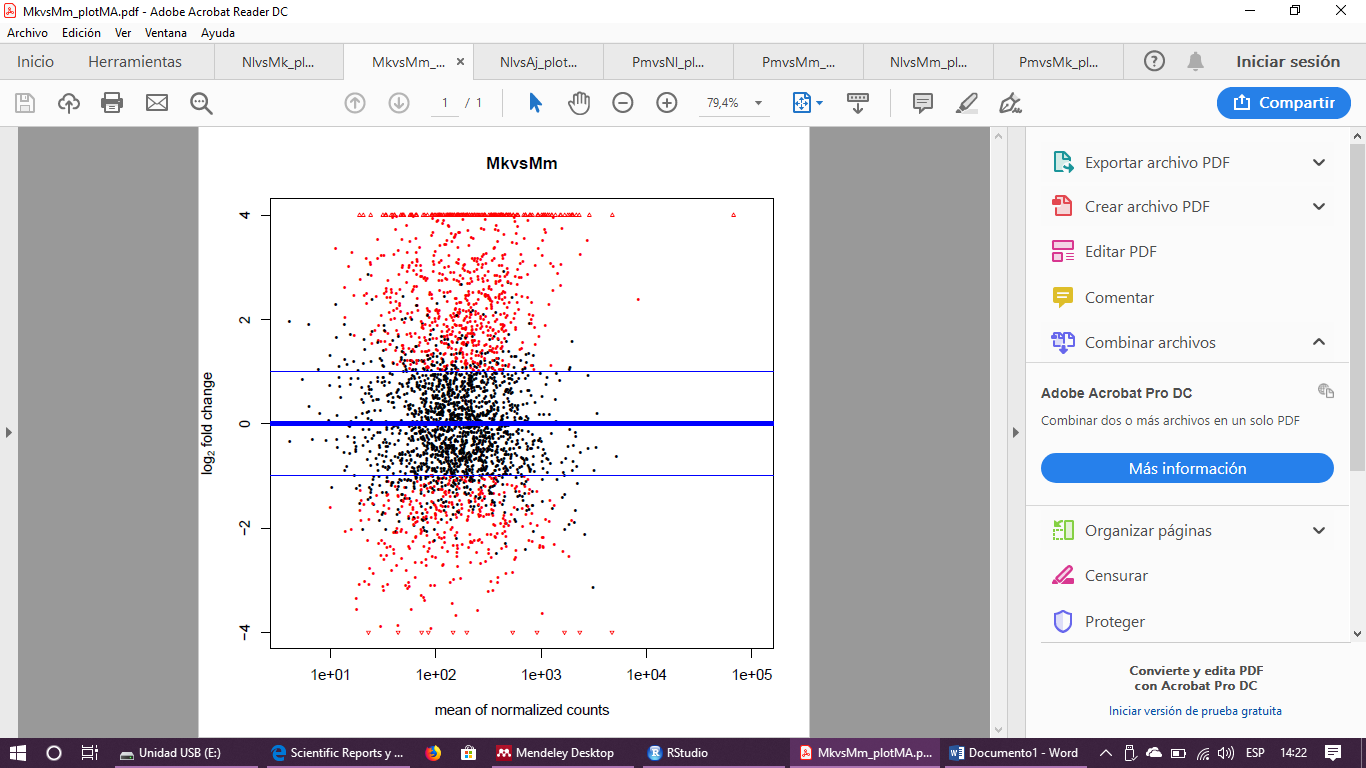


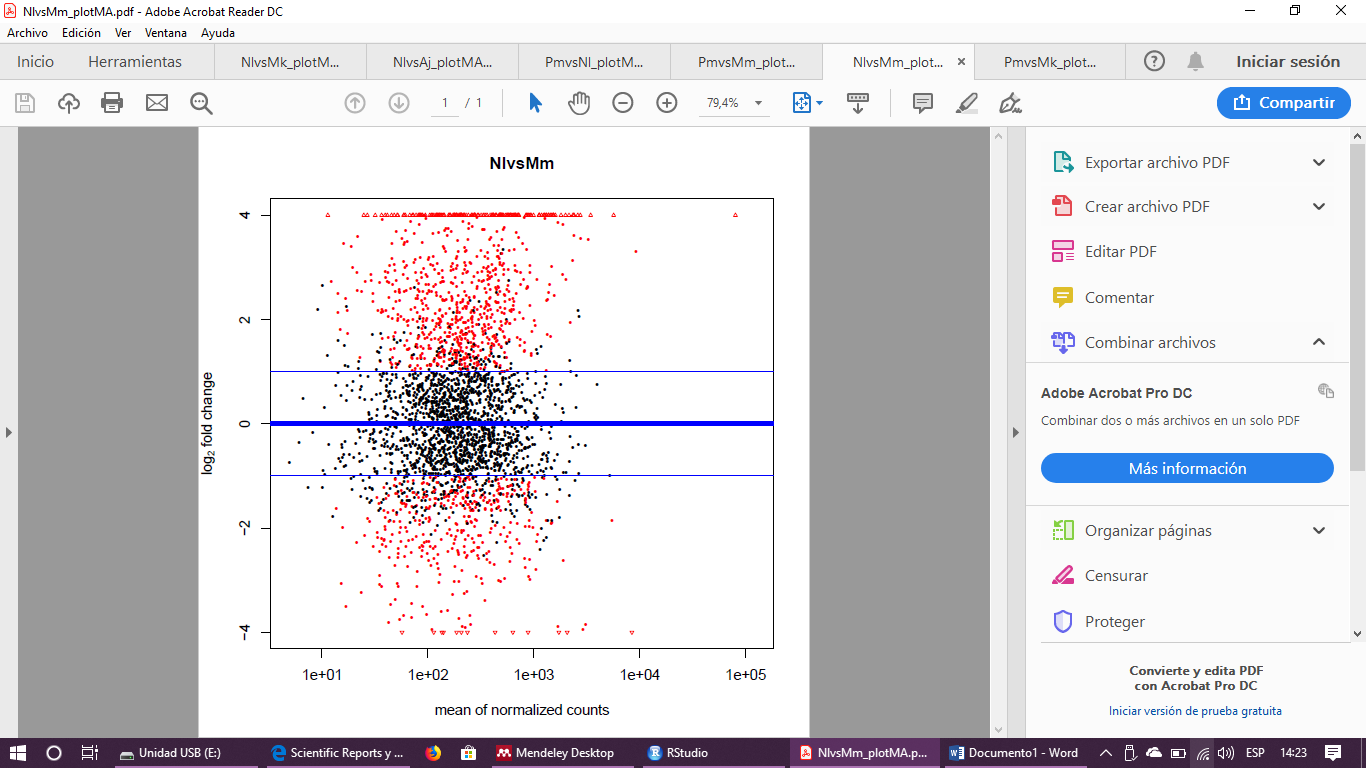


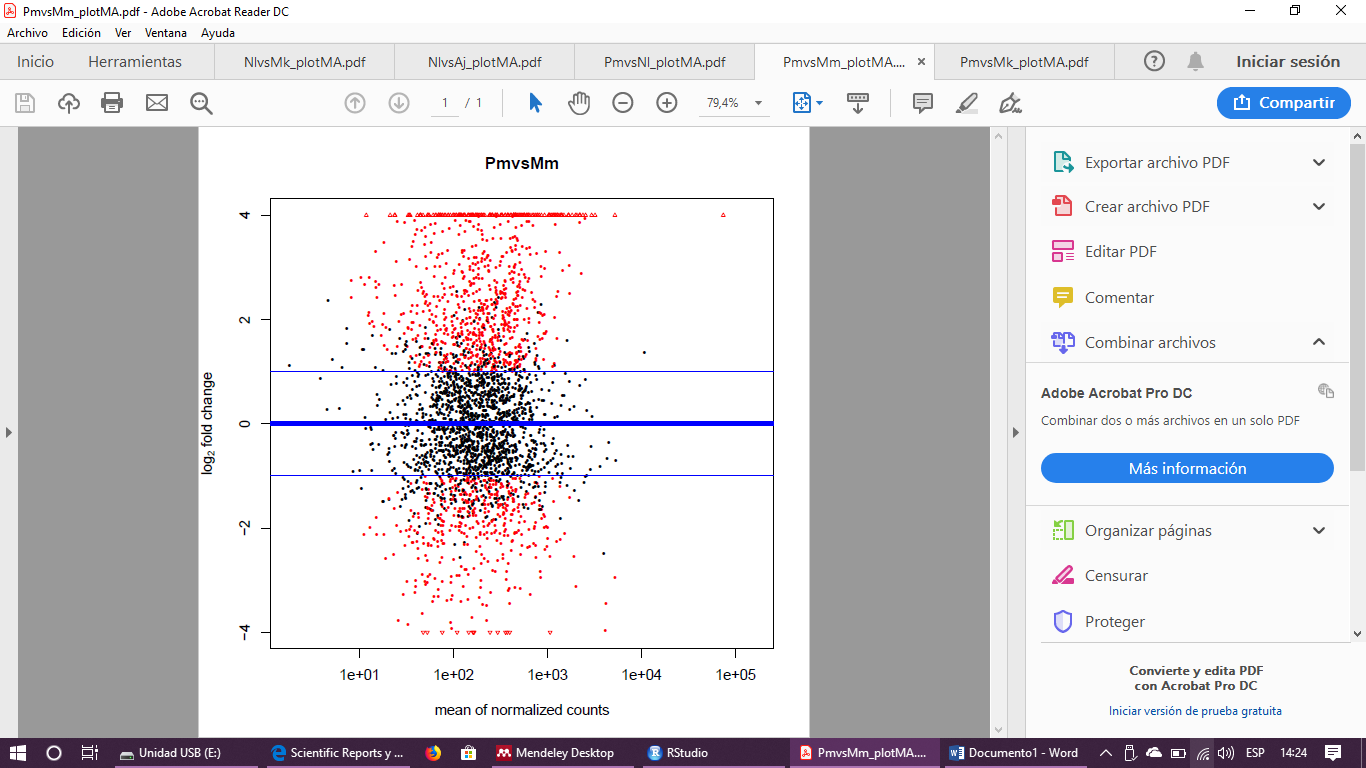


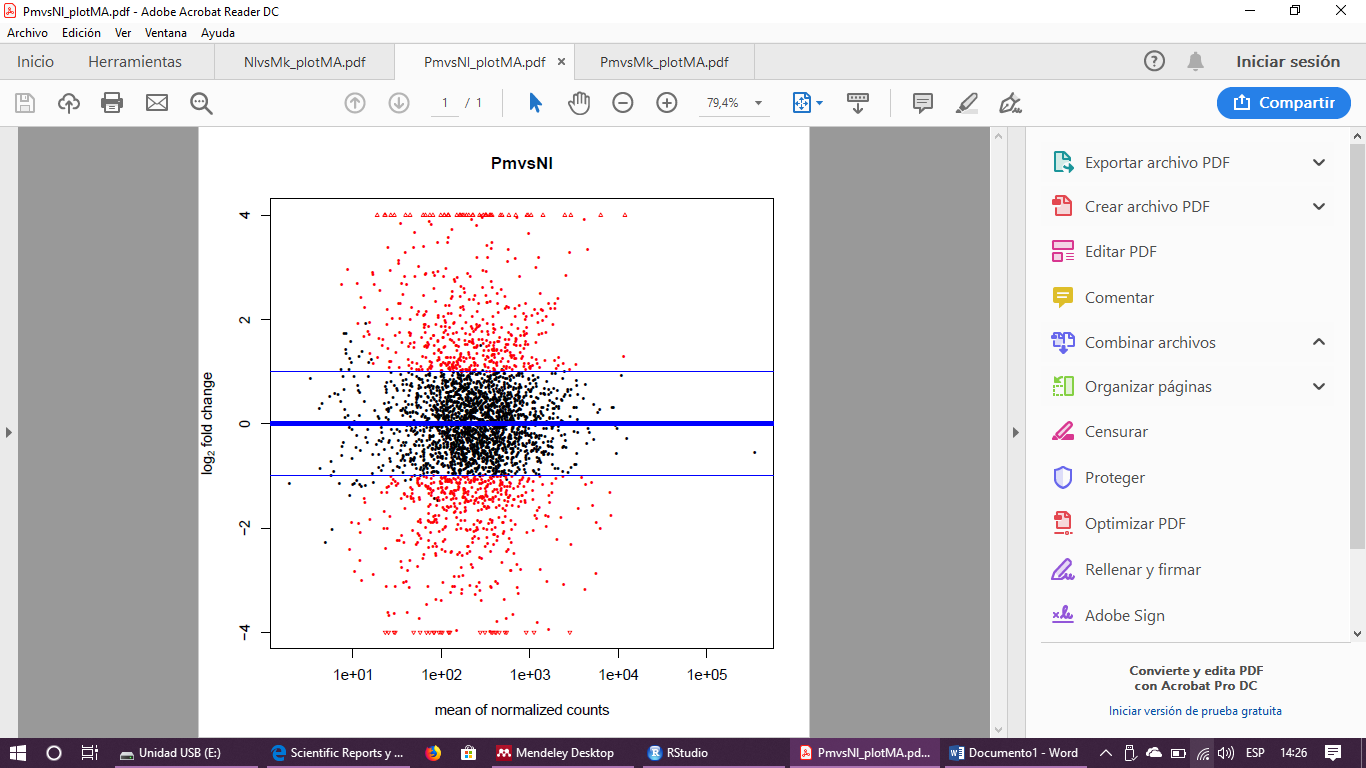


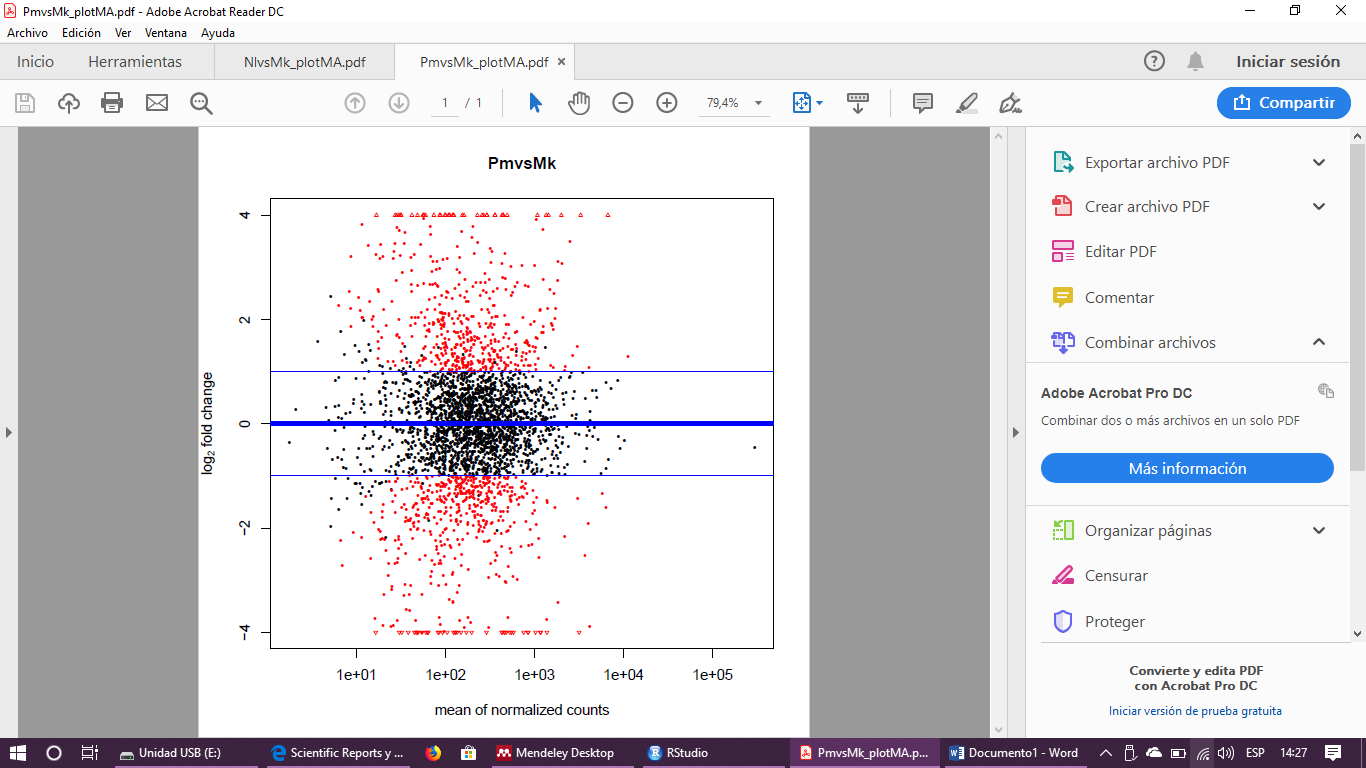


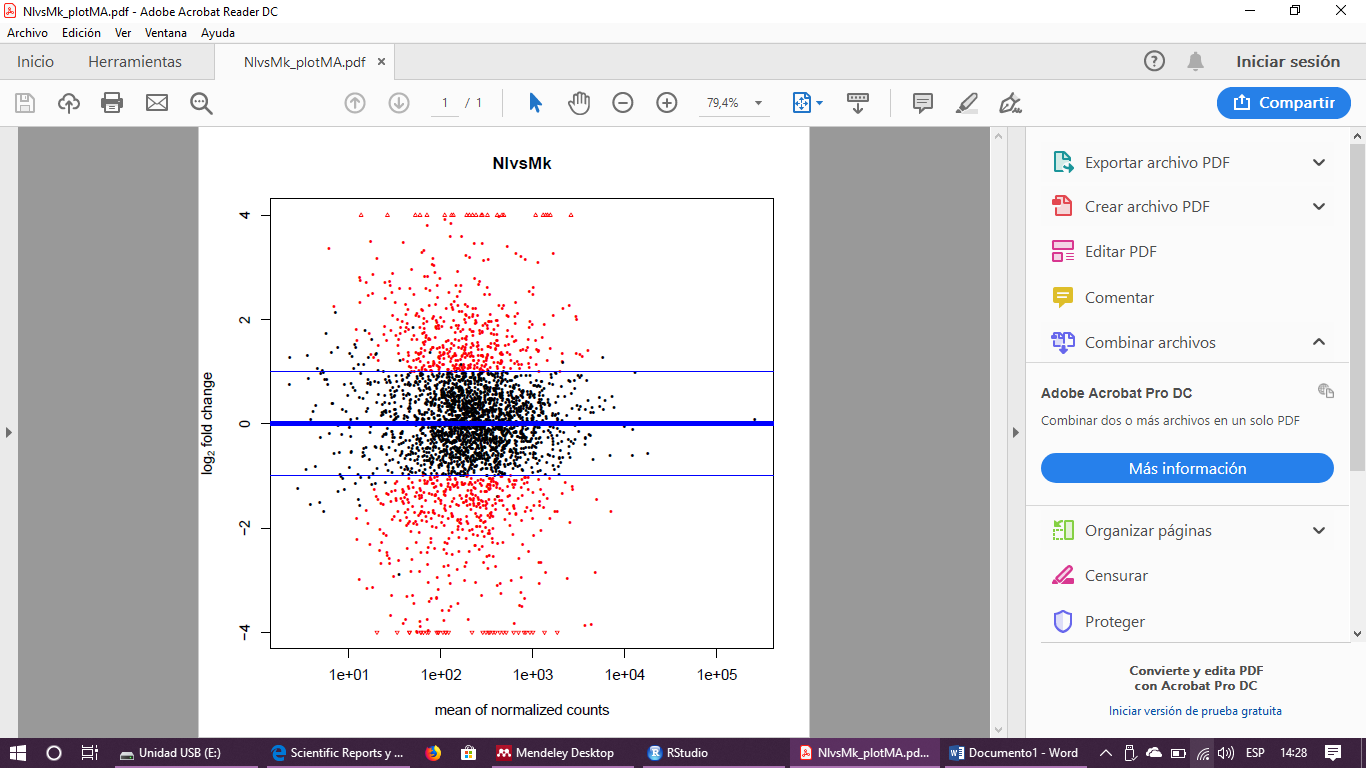


**Supplementary table S3.** Upregulated single copy orthologues in *Artibeus jamaicensis* with respective log2 Fold Change values for the five DE comparisons between insectivorous species and *A. jamaicensis* (*p < 0.01).*

|  |  |  |  |  | **Aj vs Mm** | **Aj vs Mk** | **Aj vs Nl** | **Aj vs Pm** |
| --- | --- | --- | --- | --- | --- | --- | --- | --- |
| **Gene symbol** | **Gene Name** | **Molecular Function** | **Biological Process** | **Mean of reads counts** | **Log2fc** | **Log2fc** | **Log2fc** | **Log2fc** |
| Miox | Inositol oxygenase | Oxidoreductase activity  (GO:0016491) | Carbohydrate metabolic process  (GO:0005975) | 72,283 | 8.62 | 11.25 | 11.08 | 11.90 |
| Acod | Acyl-CoA desaturase | Stearoyl-CoA 9-desaturase  (GO:0004768) | Long-chain fatty -acyl-CoA biosynthetic process  (GO:0035338) | 27,576 | 6.04 | 9.68 | 8.21 | 6.79 |
| Tkt | Transketolase | Oxidoreductase activity  (GO:0016491) | Monosaccharide metabolic process (GO:0005996) | 12,976 | 4.33 | 4.70 | 3.92 | 4.41 |
| Ck054 | Ester hydrolase | Hydrolase activity  (GO:0016788) | -- | 9,335 | 4.11 | 4.20 | 3.66 | 3.89 |
| Dhi1 | Corticosteroid 11-beta-dehydrogenase isozyme 1 | Oxidoreductase activity, (go:0016616)  Steroid binding  (GO:0005496) | Glucocorticoid biosynthetic process  (GO:0006704) | 4,866 | 3.61 | 8.05 | 1.69 | 2.00 |
| Kcnj8 | Atp-sensitive inward rectifier potassium channel | ATP-activated inward rectifier potassium channel activity  (GO:0015272) | Potassium ion transport  (GO:0006813) | 3,246 | 4.94 | 2.53 | 5.65 | 5.38 |
| St17b | Serine/threonine-protein kinase | ATP binding  (GO:0005524) | Apoptotic process (GO:0006915) | 1,754 | 3.60 | 2.52 | 4.12 | 4.25 |
| Orml3 | Orm1-like protein | -- | Ceramide metabolic process  (GO:0006672) | 1,509 | 5.08 | 4.54 | 2.49 | 3.32 |
| Cisy | Citrate synthase, mitochondrial | Transferase activity, transferring acyl groups  (GO:0016746) | Carbohydrate metabolic process  (GO:0005975) | 1,492 | 2.87 | 1.82 | 2.44 | 1.51 |
| G6pd | Glucose-6-phosphate1-dehydrogenase | Glucose-6-phosphate dehydrogenase activity  (GO:0004345) | Glucose metabolic process  (GO:006006) | 1,171 | 2.95 | 2.79 | 3.72 | 4.30 |
| Rcn1 | Reticulocalbin-1 | Calicum ion binding (GO:0005509) | -- | 1,119 | 5.01 | 2.28 | 3.87 | 3.20 |
| Abcc9 | Atp binding cassette subfamily c member 9 | ATPase activity  (GO:0005524) | Potassium ion transport (GO:0006813) | 1,095 | 3.96 | 2.10 | 2.35 | 2.43 |
| Rbns5 | Rabenosyn-5 | Zinc ion binding  (GO:0008270) | Protein transport (GO:0015031) | 1,062 | 2.77 | 1.75 | 1.68 | 3.12 |
| Scot1 | Succinyl-coa:3-ketoacid coenzyme a transferase 1, mitochondrial | 3-oxoacid CoA-transferase activity  (GO:0008060) | Positive regulation of insulin secresion  (GO:0035774) | 951 | 5.08 | 6.74 | 3.22 | 4.03 |
| Itm2c | Integral membrane protein 2c | Amyloid-beta binding  (G0:0001540) | Glycoprotein biosynthetic process  (GO:0009100) | 766 | 2.85 | 4.87 | 5.06 | 4.21 |
| 3bp5l | Sh3 domain-binding protein 5-like | Protein kinase inhibitor activity  (GO:0004860) | Intracellular signal transduction  (GO:0035556) | 683 | 3.20 | 1.46 | 2.03 | 1.75 |
| Amgo3 | Amphoterin-induced protein 3 | -- | Positive regulation of synapse assembly  (GO:00051965) | 610 | 4.67 | 2.94 | 2.11 | 1.29 |
| Tbl2 | Transducin beta-like protein | Phosphoprotein binding  (GO:0019901) | Cellular response to glucose starvation  (G0:0042149) | 533 | 2.74 | 1.43 | 1.01 | 1.25 |
| Snf5 | Swi/snf-related matrix-associated actin-dependent regulator of chromatin subfamily b | Protein binding  (GO:0005515) | Regulation of transcription by RNA polymerase II  (GO:0006357) | 505 | 2.45 | 1.65 | 2.52 | 1.93 |
| Nsun4 | Nop2/sun rna methyltransferase | Methyltransferase activity  (GO:0008168) | rRNA methylation  (GO:0031167) | 480 | 3.57 | 1.18 | 1.07 | 1.73 |
| Tad2a | Transcriptional adapter 2-alpha | Transcription coactivator activity  (GO:0003713) | Regulation of transcription by RNA polymerase II  (GO:0006357) | 467 | 2.71 | 1.50 | 1.29 | 1.94 |
| Nde1 | Nuclear distribution protein | Microtubule binding  (GO:0008017) | Mitotic nuclear division  (GO:0140014); | 442 | 2.41 | 4.13 | 4.55 | 4.56 |
| Hspb1 | Heat shock protein beta | Heat shock protein  (GO:0031072) | Immune system process  (GO:0002376) | 399 | 3.31 | 4.34 | 3.51 | 2.44 |
| Cybr1 | Cytochrome b reductase 1 | Oxidoreductase activity  (GO:0016491) | Cellular iron ion homeostasis  (GO:0006879) | 349 | 3.87 | 3.81 | 2.33 | 3.02 |
| Mlkl | Mixed lineage kinase domain-like pseudokinase | Protein kinase activity  (GO:0004672) | Protein phosphorylation  (GO: 0006468) | 307 | 3.26 | 3.14 | 3.89 | 2.52 |
| Ubtd2 | Ubiquitin domain | Ubiquitin binding  (GO:0043130) | -- | 280 | 2.82 | 2.19 | 1.62 | 2.56 |
| C56d1 | Cytochrome b561 domain | Metal ion binding  (GO:0055114) | Oxidation reduction process  (GO:0055114) | 258 | 2.71 | 3.35 | 6.16 |  |

**Supplementary Table S4.** Annotation of upregulated single copy orthologues in *Mormoops megalophylla* with respective log2 Fold Change values (*p < 0.01)* and mean reads counts*.* DE comparisons were performed against *Artibeus jamaicensis, Myotis keaysi, Nyctinomops laticaudatus* and *Peropteryx macrotis.*

|  |  |  |  |  | **Mm vs Aj** | **Mm vs Mk** | **Mm vs Nl** | **Mm vs Pm** |
| --- | --- | --- | --- | --- | --- | --- | --- | --- |
| **Gene symbol** | **Gene Name** | **Molecular Function** | **Biological Process** | **Mean of reads counts** | **Log2fc** | **Log2fc** | **Log2fc** | **Log2fc** |
| Homez | Homeobox and leucine zipper protein | DNA-binding transcription factor activity (GO:0003700)  Transcription corepressor activity  (GO:0003714) | Negative regulation of transcription by rna polymerase ii  (GO:0000122) | 9,539 | 10.25 | 11.10 | 11.79 | 11.35 |
| At2c1 | Calcium-transporting atpase type 2c member | Calcium ion transmembrane transporter activity  (GO:0015085) | Cellular calcium ion homeostasis  (GO:0006874) | 6,688.67 | 4.75 | 4.67 | 4.56 | 4.90 |
| Cog2 | Conserved oligomeric golgi complex subunit 2 | Protein transporter activity  (GO:0008565) | Golgi organization  (GO:0007030) | 4,107.33 | 5.08 | 5.29 | 5.09 | 4.85 |
| Mk07 | Mitogen-activated protein kinase 7 | Protein kinase activity  (GO:0004672) | Regulation of gene expression  (GO:0010468) | 3,367.33 | 8.44 | 8.94 | 9.56 | 9.31 |
| M3k1 | Mitogen-activated protein kinase kinase kinase 1 | Protein serine/threonine kinase activity  (GO:0004674) | Activation of protein kinase activity  (GO:0032147) | 3,171.67 | 3.66 | 3.25 | 3.55 | 3.94 |
| Nostn | Nostrin | DNA binding (GO:0003677) | Negative Regulation of transcription  (GO:0045892) | 3,123.67 | 7.58 | 6.90 | 5.71 | 7.27 |
| Ant3 | Antithrombin-III | Endopeptidase inhibitor activity (GO:0004866)  Protease binding  (GO:0002020 | Cellular protein metabolic process  (GO:0044267) | 2,928.33 | 11.70 | 11.84 | 10.61 | 11.46 |
| Frat2 | Frat2, wnt-signaling pathway regulator | -- | -- | 2,898.33 | 5.45 | 5.25 | 6.59 | 4.87 |
| Nbn | Nibrin | ATP-dependent DNA helicase activity  (GO:0004003) | Signal transduction in response to DNA damage  (GO:0042770) | 2,739.00 | 3.90 | 4.47 | 3.62 | 3.86 |
| Ube3b | Ubiquitin-protein ligase | Ubiquitin-protein transferase activity  (GO:0004842) | Protein polyubiquitination  (GO:0000209) | 2,612.33 | 3.00 | 4.06 | 3.12 | 4.10 |
| Mph6 | M-phase phosphoprotein | RNA binding  (GO:0003723) | Maturation of 5.8s rrna  (GO:0000460) | 2,607.33 | 6.17 | 7.18 | 6.59 | 6.69 |
| Stabp | Stam-binding protein | Thiol-dependent ubiquitin-specific protease activity  (GO:0004843) | Protein k63-linked deubiquitination  (GO:0070536) | 2,386.00 | 4.94 | 4.67 | 4.83 | 5.28 |
| Etv6 | Transcription factor etv6 | RNA polymerase II transcription factor activity  (GO:0000981) | Transcription by RNA polymerase II (GO:0006366) | 2,233.67 | 3.64 | 4.39 | 3.83 | 4.89 |
| Bcl9l | B-cell cll/lymphoma 9-like | -- | -- | 2,178.67 | 3.49 | 3.75 | 2.76 | 5.20 |
| Ipo11 | Importin-11;ipo11 | Protein transporter activity  (GO:0008565) | Protein import into nucleus  (GO:0006606) | 2,152.33 | 4.60 | 4.79 | 4.93 | 4.77 |
| Palld | Palladin | Protein binding  (GO:0005515) | Cell-cell adhesion via plasma-membrane adhesion molecules  (GO:0098742) | 2,015.33 | 5.47 | 4.92 | 6.56 | 5.58 |
| Lhfp | Lhfpl tetraspan subfamily member 1 protein | Molecular function  (GO:0003677) | -- | 1,856.00 | 5.14 | 7.36 | 7.12 | 7.89 |
| Suv3 | ATP-dependent RNA helicase | ATP-dependent RNA helicasa  (GO:0004004) | Mitochondrial mRNA catabolic process (GO:0035946) | 1,817.33 | 4.43 | 3.96 | 4.29 | 4.63 |
| Ap4b1 | Ap complex subunit beta | -- | Vesicle-mediated transport  (GO:0016192) | 1,800.33 | 4.61 | 4.91 | 4.36 | 5.44 |
| K1328 | Protein hinderin | -- | -- | 1,750.60 | 8.57 | 8.91 | 8.89 | 8.97 |
| Tm230 | Transmembrane protein | -- | Synaptic vesicle  (GO:0048489) | 1,718.33 | 4.18 | 3.01 | 4.03 | 2.94 |
| Smyd4 | Set and mynd domain-containing protein 4 | Methyltransferase  (GO:0008168) | -- | 1,688.67 | 4.87 | 8.34 | 8.43 | 7.43 |
| Tmcc2 | Transmembrane and coiled-coil domains protein | -- | -- | 1,687.67 | 9.59 | 9.34 | 8.20 | 8.14 |
| Ubtd1 | Ubiquitin domain-containing protein | Ubiquitin-protein transferase activity  (GO:0004842) | -- | 1,579.33 | 4.80 | 5.80 | 5.00 | 5.11 |
| Bl1s4 | Biogenesis of lysosome-related organelles complex 1 subunit 4 | -- | Intracellular transport  (GO:0046907) | 1,518.67 | 5.68 | 5.27 | 5.20 | 4.98 |
| Cg026 | Uncharacterized protein | -- | -- | 1,515.00 | 4.27 | 3.66 | 4.73 | 4.69 |
| Rfc5 | Replication factor c | DNA binding  (GO:0003677)  DNA-dependent atpase activity  (GO:0008094) | DNA biosynthetic process  (GO:0071897); | 1,201.00 | 6.24 | 5.41 | 5.26 | 4.60 |

**Supplementary Table S5.** Annotation of upregulated single copy orthologues in *Myotis keaysi* with respective log2 Fold Change values (*p < 0.01)* and mean reads counts*.* DE comparisons were performed against *Artibeus jamaicensis, Mormoops megalophylla, Nyctinomops laticaudatus* and *Peropteryx macrotis.*

|  |  |  |  |  | **Mk vs Aj** | **Mk vs Mm** | **Mk vs Nl** | **Mk vs Pm** |
| --- | --- | --- | --- | --- | --- | --- | --- | --- |
| **Gene symbol** | **Gene Name** | **Molecular Function** | **Biological Process** | **Mean of reads counts** | **log2FC** | **log2FC** | **log2FC** | **log2FC** |
| Optn | Optineurin | Polyubiquitin modification-dependent protein binding  (GO:0031593) | Autophagy  (GO:0006914) | 4,518.67 | 4.52 | 5.16 | 4.10 | 3.50 |
| Rtp4 | Receptor-transporting protein | G-protein coupled receptor binding  (GO:0001664) | Protein insertion into membrane  (GO:0051205) | 2,436.67 | 3.21 | 5.64 | 5.96 | 2.57 |
| Ce85l | Centrosomal protein | -- | -- | 2,203.00 | 3.64 | 2.34 | 4.34 | 3.73 |
| Xylk | Glycosaminoglycan xylosylkinase | Phosphotransferase activity. alcohol group as acceptor  (GO:0016773) | Protein phosphorylation  (GO:0006468) | 1,355.67 | 1.52 | 2.57 | 3.15 | 1.04 |
| Ci064 | Queuosine salvage protein | -- | tRNA modification  (GO:0006400) | 1,001.67 | 1.88 | 2.36 | 3.27 | 2.70 |
| Akib1 | Ankyrin repeat and ibr domain-containing protein 1 | Ubiquitin protein ligase activity  (GO:0061630) | Positive regulation of proteasomal ubiquitin-dependent protein catabolic process  (GO:0032436) | 943.33 | 1.26 | 2.39 | 1.40 | 1.21 |
| Ppbt | Alkaline phosphatase | Alkaline phosphatase activity  (GO:0004035) | Dephosphorylation  (GO:0016311) | 688.00 | 2.32 | 2.41 | 1.21 | 1.91 |
| Fa84b | Protein fam84b | -- | -- | 482.00 | 1.72 | 2.28 | 2.10 | 1.85 |
| Timd4 | T-cell immunoglobulin and mucin domain | -- | -- | 372.33 | 1.31 | 2.33 | 1.41 | 1.47 |
| Tsn15 | Tetraspanin-15 | Enzyme binding  (GO:0019899) | Cell surface receptor signaling pathway  (GO:0007166) | 336.67 | 3.36 | 2.47 | 2.08 | 3.26 |
| Pgap2 | Post-gpi attachment to proteins factor 2 | Protein transporter activity  (GO:0008565) | Gpi anchor biosynthetic process  (GO:0006506) | 363.33 | 1.32 | 3.05 | 1.92 | 2.63 |
| Fwch1 | Flywch-type zinc finger | Metal ion binding  (GO:0046872) | -- | 387.00 | 3.11 | 2.31 | 1.88 | 3.47 |
| Taco1 | Translational activator of cytochrome c oxidase 1 | -- | Regulation of translation  (GO:0006417) | 339.33 | 1.97 | 3.46 | 1.31 | 1.20 |
| Par1 | Solute carrier family 52 | G-protein coupled receptor activity  (GO:0004930) | Cell surface receptor signaling pathway  (GO:0007166) | 249.67 | 2.12 | 3.10 | 1.06 | 3.40 |
| Lrc20 | Leucine-rich repeat-containing protein | -- | -- | 249.33 | 4.39 | 5.17 | 4.86 | 5.52 |
| Dph5 | Diphthine methyl ester synthase | Diphthine synthase activity  (GO:0004164) | Peptidyl-diphthamide biosynthetic process from peptidyl-histidine  (GO:0017183) | 231.33 | 1.74 | 2.57 | 1.27 | 1.70 |
| Cg025 | Upf0415 protein | -- | -- | 182.67 | 1.18 | 3.34 | 4.70 | 1.93 |
| Srbs2 | Sorbin and sh3 domain-containing protein 2 | Nucleic acid binding  (GO:0003676) | Biological process actin filament organization  (GO:0007015) | 108.00 | 4.24 | 4.00 | 2.82 | 1.92 |
| Arg28 | Rho guanine nucleotide exchange factor | Rho guanyl nucleotide exchange factor activity  (GO:0005089) | Regulation of Rho protein signal transduction  (GO:0035023) | 75.00 | 5.93 | 3.13 | 2.97 | 4.41 |

**Supplementary Table S6.** Annotation of upregulated single copy orthologues in *Nyctinomopos laticaudatus* with respective log2 Fold Change values (*p < 0.01)* and mean reads counts*.* DE comparisons were performed against *Artibeus jamaicensis, Mormoops megalophylla, Myotis keaysi* and *Peropteryx macrotis.*

|  |  |  |  |  | **Nl vs**  **Aj** | **Nl vs Mm** | **Nl vs Mk** | **Nl vs Pm** |
| --- | --- | --- | --- | --- | --- | --- | --- | --- |
| **Gene Symbol** | **Gene Name** | **Molecular Function** | **Biological Process** | **Mean of reads counts** | **log2FC** | **log2FC** | **log2FC** | **log2FC** |
| ABHEB | Protein ABHD14B | Hydrolase activity  (GO:0016787) | Positive regulation of transcription from RNA polymerase II promoter  (GO:0045944) | 8,794.67 | 6.41 | 3.86 | 1.45 | 3.33 |
| LCAT | Phosphatidylcholine-sterol acyltransferase | Phospholipase activity  (GO:0004620); | Cholesterol metabolic process  (GO:0008203) | 5,072.33 | 5.02 | 5.26 | 2.98 | 4.34 |
| TXNIP | Thioredoxin-interacting protein; | Enzyme inhibitor  (GO:0004857) | Cell cycle  (GO:0007049) | 4,756.33 | 1.88 | 3.89 | 1.90 | 2.84 |
| F149A | Protein FAM149A | -- | -- | 2,136.00 | 2.84 | 2.59 | 4.17 | 2.12 |
| -- | Unannotated | -- | -- | 1,905.67 | 4.50 | 7.77 | 6.11 | 4.50 |
| NHSL1 | NHS-like protein | -- | Cell differentiation  (GO:0030154) | 1,542.00 | 1.83 | 2.65 | 2.31 | 1.85 |
| MBP | Myelin basic protein; | Molecular function structural constituent of myelin sheath  (GO:0019911) | Immune response  (GO:0006955) | 1,454.00 | 7.00 | 2.09 | 2.59 | 5.79 |
| TP8L1 | Tumor necrosis factor alpha-induced protein 8-like protein | -- | Negative regulation of TOR signaling  (GO:0032007) | 1,224.67 | 2.25 | 4.13 | 8.58 | 1.83 |
| FGRL1 | Fibroblast growth factor receptor-like | Fibroblast growth factor-activated  (GO:0005007) | Negative regulation of cell proliferation  (GO:0008285) | 1,008.00 | 1.70 | 2.66 | 1.17 | 1.72 |
| MIPEP | Mitochondrial intermediate peptidase | Metalloendopeptidase activity  (GO:0004222) | Peptide metabolic process  (GO:0006518) | 970.33 | 2.41 | 2.45 | 1.64 | 1.31 |
| -- | Unannotated | -- | -- | 825.33 | 1.94 | 2.27 | 2.74 | 1.39 |
| DCUP | Uroporphyrinogen decarboxylase | Uroporphyrinogen decarboxylase activity  (GO:0004853) | Protoporphyrinogen IX biosynthetic process  (GO:0006782) | 742.67 | 1.80 | 2.15 | 1.25 | 1.10 |
| -- | Unannotated | -- | -- | 685.00 | 1.17 | 4.42 | 2.77 | 2.73 |
| ZN217 | Zinc finger protein | Transcriptional repressor activity, RNA polymerase II core promoter  (GO:0001078) | Negative regulation of transcription  (GO:0000122) | 606.33 | 1.95 | 3.26 | 1.75 | 1.32 |
| DGAT1 | Diacylglycerol O-acyltransferase | Transferase activity, transferring acyl groups  (GO:0016747) | Triglyceride metabolic process  (GO:0006641) | 559.67 | 1.21 | 2.82 | 1.90 | 1.76 |
| TAF8 | Transcription initiation factor | -- | Positive regulation of transcription  (GO:0045893) | 524.67 | 1.46 | 3.02 | 2.41 | 1.39 |
|  | Unannotated | -- | -- | 574.00 | 1.58 | 2.47 | 2.36 | 1.31 |
| IDS | Iduronate 2-sulfatase | Hydrolase activity, acting on ester bonds  (GO:0016788) | Glycosaminoglycan catabolic process  (GO:0006027) | 452.00 | 2.99 | 2.16 | 1.53 | 1.14 |
| VASN | Vasorin; | Cadherin binding involved in cell-cell adhesion  (GO:0098641) | Cell surface receptor signaling pathway  (GO:0007166) | 441.00 | 1.61 | 2.29 | 1.73 | 3.91 |
| MGME1 | Mitochondrial genome maintenance exonuclease | Deoxyribonuclease activity  (GO:0004536) | DNA biosynthetic process  (GO:0071897) | 389.33 | 3.31 | 4.21 | 3.02 | 4.12 |

**Supplementary Table S7.** Annotation of upregulated single copy orthologues in *Peropteryx macrotis* with respective log2 Fold Change values (*p < 0.01)* and mean reads counts*.* DE comparisons were performed against *Artibeus jamaicensis, Mormoops megalophylla, Myotis keaysi* and *Nyctinomops laticaudatus.*

|  |  |  |  |  | **Pm vs Aj** | **Pm vs**  **Mm** | **Pm vs Mk** | **Pm vs Nl** |
| --- | --- | --- | --- | --- | --- | --- | --- | --- |
| **Gene Symbol** | **Gene Name** | **Molecular Function** | **Biological Process** | **Mean of reads counts** | **log2FC** | **log2FC** | **log2FC** | **log2FC** |
| Est1 | Liver carboxylesterase | Carboxylic ester hydrolase activity  (GO:0052689) | -- | 11,064.67 | 1.39 | 3.97 | 1.61 | 1.32 |
| Echb | Trifunctional enzyme subunit beta | Acetyltransferase activity  (GO:0016407) | Fatty acid catabolic process  (GO:0009062) | 5,156.33 | 1.36 | 3.12 | 1.43 | 1.64 |
| Cu025 | -- | -- | -- | 3,017.00 | 4.35 | 5.01 | 3.75 | 3.94 |
| Ccd97 | Coiled-coil domain-containing protein 97 | -- | -- | 1,317.00 | 2.30 | 3.17 | 2.99 | 2.79 |
| Adprh | [protein adp-ribosylarginine] hydrolase | Gtpase activator activity  (GO:0005096) | Cellular protein modification process  (GO:0006464) | 1,206.33 | 3.39 | 2.51 | 1.41 | 3.15 |
| Vwce | Von willebrand factor C and EGF domain-containing protein | Calcium ion storage activity  (GO:0005509) | Cellular response to virus  (GO:0098586) | 1,033.33 | 3.18 | 3.44 | 2.40 | 2.81 |
| Cf132 | Uncharacterized protein | -- | -- | 1,025.67 | 2.82 | 4.42 | 3.72 | 2.50 |
| Sco2 | Protein sco2 homolog | Oxidoreductase activity  (GO:0016491) | Cation transport  (GO:0006812) | 1,020.00 | 4.89 | 4.43 | 2.73 | 2.82 |
| Tatd2 | TatD DNase Domain | DNA metabolic process  (GO:0055132) | -- | 1,020.00 | 2.04 | 3.16 | 1.36 | 1.08 |
| C2cd3 | C2 domain-containing protein 3 | -- | Protein localization to centrosome  (GO: 0071539) | 983.33 | 1.76 | 2.43 | 1.97 | 5.56 |
| Lypla1 | Lysophospholipase-like protein | Carboxylic ester hydrolase activity  (GO:0052689) | Protein depalmitoylation  (GO:0002084) | 929.00 | 5.30 | 4.14 | 3.02 | 3.74 |
| Slc35g1 | Solute carrier family | -- | Calcium ion export across plasma membrane  (GO:1990034) | 915.33 | 1.80 | 2.99 | 1.39 | 2.20 |
| Wfdc1 | Wap four-disulfide core domain protein 1 | -- | Cell growth  (GO:0016049) | 825.67 | 3.49 | 5.56 | 2.35 | 4.18 |
| Dps1 | Decaprenyl-diphosphate synthase subunit 1 | Transferase activity, transferring acyl groups  (GO:0016746) | Metabolic process  (GO:0008152) | 663.67 | 4.11 | 4.25 | 3.92 | 4.01 |
| LAS1L | Ribosomal biogenesis protein | -- | Maturation of 5.8s rrna  (GO:0000460) | 644.33 | 2.55 | 3.25 | 1.66 | 1.28 |
| Ston2 | Stonin-2 | -- | Endocytosis  (GO:0006897) | 529.67 | 1.11 | 3.46 | 1.29 | 1.85 |
| SLC19A1 | Folate transporter | Amide binding  (GO:0033218) | Transmembrane transport  (GO:0055085) | 483.67 | 4.81 | 3.33 | 2.07 | 1.59 |
| Int7 | Integrator complex subunit 7 | -- | snRNA3'-end processing  (GO:0034472) | 245.00 | 1.46 | 2.60 | 1.30 | 1.28 |
| Ccd34 | Coiled-coil domain-containing protein | -- | -- | 238.00 | 7.07 | 3.05 | 1.84 | 2.93 |
| Cul7 | Cullin-7 | Microtubule cytoskeleton organization  (GO:0000226) | -- | 175.67 | 4.19 | 2.67 | 2.68 | 5.38 |
